# Strain-Distinct α‑Synuclein and Tau Cross-Seeding Uncovered by Correlative Approach with Optical Photothermal Infrared Sub-Micron Imaging

**DOI:** 10.1021/jacs.5c02811

**Published:** 2025-07-29

**Authors:** Xiaoni Zhan, Wen Li, Eric Hatterer, Jean-Philippe Courade, Kristin Piché, Oxana Klementieva, Jia-Yi Li

**Affiliations:** † Department of Forensic Genetics and Biology, School of Forensic Medicine, 26488 China Medical University, Shenyang 110122, China; ‡ Neural Plasticity and Repair Unit, Department of Experimental Medical Science, Wallenberg Neuroscience Center, 5193 Lund University, BMC A10, Lund 22184, Sweden; § Health Sciences Institute, Key Laboratory of Major Chronic Diseases of Nervous System of Liaoning Province, China Medical University, Shenyang 110122, China; ∥ 213627 Light Chain Bioscience, Chemin du pré-Fleuri 15, Plan-les-Ouates 1228, Switzerland; ⊥ Discoveric Bio Alpha, Bahnhofstrasse 1, Pfäffikon 8808, Switzerland; # StressMarq Biosciences Inc., 118-1537 Hillside Avenue, Victoria BC V8T 2C1, British Columbia; ¶ Medical Microspectroscopy Research Group, Department of Experimental Medical Science, Lund University, BMC B10, Lund 22180, Sweden; ∇ NanoLund, Lund University, Lund 22100, Sweden

## Abstract

The co-occurrence of α-synuclein (αSyn) and Tau in synucleinopathies and tauopathies suggests a complex interplay between these proteins. Their cross-seeding enhances fibrillization, leading to the formation of diverse amyloid-specific structures enriched with β-sheets, which may influence their biological functions. However, existing tools cannot differentiate structural polymorphs directly in cells, as conventional microscopic approaches have limitations in providing structural insights into aggregates. As a result, a structurally relevant characterization of amyloids in their native cellular environment has not yet been achieved. In this study, we characterize the structural rearrangements of newly formed αSyn inclusions cross-seeded by different αSyn and Tau preformed fibrils (PFFs) directly in cells, using a correlative approach that combines submicron optical photothermal infrared (O-PTIR) microspectroscopy and confocal microscopy. We found that hybrid PFFs synthesized from αSyn, and two Tau isoforms (Tau3R and Tau4R) exhibit variations in αSyn and Tau composition. Specifically, structural polymorphs composed of αSyn and Tau3R exhibit the highest β-sheet content and most potent seeding potency, leading to enhanced phosphorylation within cellular inclusions. Importantly, we demonstrate that cellular inclusions inherit structural motifs from their donor seeds and exhibit distinct spatial and structural evolution. By providing subcellular-resolution structural imaging of amyloid proteins, our study uncovers divergent mechanisms of αSyn aggregation induced by αSyn/Tau PFFs in both mixed and hybrid formats.

## Graphical abstract

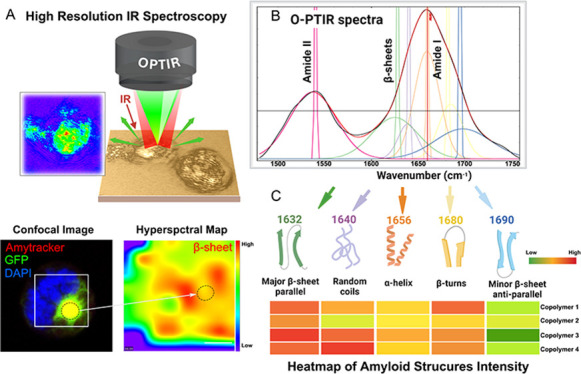

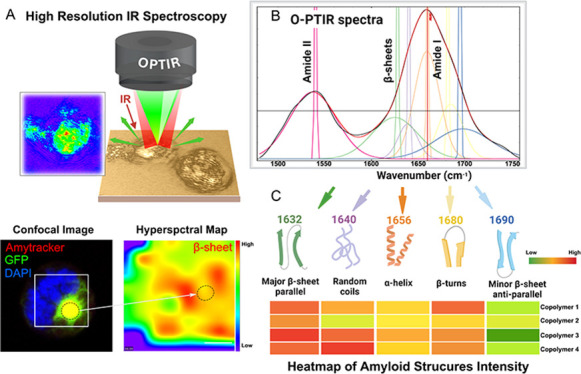

## 1 Introduction

Synucleinopathies, including Parkinson’s disease (PD), multiple system atrophy (MSA), and dementia with Lewy bodies (DLB), share similar pathological features of α-Synuclein (αSyn) deposition.^1^ Insoluble αSyn fibrils extracted from different synucleinopathies have diverse pathogenicity, implying the presence of distinct structural “strains”, which lead to pathological and clinical heterogeneity.^2,3^ The microtubule-associated protein, Tau, can undergo hyperphosphorylation and aggregation forming neurofibrillary tangles in tauopathies.^4,5^ Accumulating evidence confirmed codeposits of αSyn and Tau in the Lewy bodies from the patients of PD, Frontotemporal lobar degeneration (FTLD), and also in Alzheimer’s Disease (AD), suggesting a pathological interplay between them.^6,7^ Post-mortem analysis of patients diagnosed with AD highlighted the presence of αSyn deposition in 50% of patients,^8,9^ indicating a pathological continuum between PD and AD. Previous studies showed that Tau interacts with αSyn and accelerates its aggregation, inducing the formation of strains with enhanced seeding activity and neurotoxicity.^10^ Although current evidence supports αSyn and Tau cross-seeding,^6,10^ the underlying molecular mechanisms behind this are still obscure.

One common pathogenic mechanism shared between AD and PD involves the accumulation of misfolded amyloid proteins with enriched β-sheets structural motifs. As they accumulate, amyloid proteins can form various structural morphs, resulting in a heterogenetic panoply of different shaped and sized aggregates.^11,12^ Interestingly, both αSyn and Tau can form multiple oligomeric and fibrillar strains with substantial structural diversity, which may lead to a variety of morphological forms of amyloids and pathological features.^13–15^ Therefore, elucidating the amyloid structures of αSyn and Tau has great significance in comprehending their cross-seeding and structure-related pathogenic properties.^16^ However, the determination of amyloid protein structures in their native environment remains challenging. With available structure-sensitive techniques such as magnetic resonance spectroscopy (NMR), circular dichroism (CD) spectroscopy, and cryo-electron microscopy, the atomic structure of amyloid fibrils has been successfully determined.^11,17^ However, these approaches require considerable sample processing, such as separation and enrichment, making structural interpretation in terms of pathogenic relevance difficult because the complex environment in which these aggregates originate and maturate is excluded from the analysis. Intracellular protein aggregates are widely studied using fluorescent dyes or conformational antibodies.^18–21^ Fluorescence provides detailed information about the distribution of proteins but is not structure-sensitive enough to discriminate between structural morphs of the same protein. We resolved the αSyn and Tau cross-seeding properties in the present study using submicron optical photothermal infrared (O-PTIR) microspectroscopy.^22–24^ Importantly, the sensitivity of O-PTIR allows the analysis of β-sheet structures characteristic of amyloids directly on cells and tissues.^25–27^ However, a major bottleneck in the use of O-PTIR is the complexity of data interpretation. We correlated O-PTIR with confocal imaging to address this challenge, allowing for a more comprehensive structural analysis. Here, we show diverse cross-seeding effects of αSyn/Tau preformed fibrils (PFFs) on intracellular human αSyn stably expressed in cultured cells. We demonstrate the disparity of amyloid structures among the strain-specific aggregates induced by pure, hybrid, or mixed PFFs, implying their morphological and physiological heterogeneity. Finally, correlative analyses of hyperspectral and fluorescent images resolved not only β-sheet distribution at the subcellular level but also distinguished the unique structural patterns of newly formed αSyn inclusions induced by different PFF seeds. Combined with the observation of structural rearrangements and development over time, the buildup of β-sheets potentially reveals the spatial evolution of different aggregates. Altogether, our study provides insight into structural perturbations of newly formed αSyn inclusions due to cross-seeding effects from the perspective of molecular structure seeding observed in the native environment at submicron resolution.

## 2 Experimental Section

### 2.1 Characterization of PFFs

All the PFFs are available at StressMarq Biosciences (StressMarq Biosciences Inc., British Columbia), specifically, αSyn monomer (SPR-321), αSyn (SPR-322), Tau 0N3R (SPR-491), Tau 2N4R (SPR-480), αS&Tau3R (SPR-494), and αS&Tau4R (SPR-495). The detailed protocol for the preparation of PFFs is available in the Supporting Information. In summary, these PFFs are made of αSyn and Tau in pure, mixed, and hybrid forms, respectively: **Pure PFFs**: 1. αSyn (αS); 2. Tau0N3R (Tau3R); 3. Tau2N4R (Tau4R); **Mixed PFFs:** 4. αS + Tau3R; 5. αS + Tau4R; **Hybrid PFFs (αS and Tau copolymers):** 6. αS&Tau3R; 7. αS&Tau4R. For clarity and brevity, the nomenclature for the aforementioned PFFs listed in Table S1 will be used consistently throughout the manuscript.

PFFs are verified and characterized via atomic force microscopy (AFM), and immunogold transmission electron microscopy (immuno-TEM). For AFM, PFFs at 1 mg/mL are diluted in ultrapure water and 10 μL is dropped onto freshly cleaved mica (12 mm, V1; VWR Cat 103302-494). The sample is incubated on the mica at room temperature (RT) for 10 min. After, the sample is washed with ultrapure water to remove the salt, and the excess liquid is removed using a fresh Kimwipe. Samples are then imaged on a Nanosurf Core AFM using an ACSTA tip in tapping mode with a 500 mV free vibration amplitude at a Z-controller set point of 55%. The width diameters of different fibrils are analyzed with the plug-in Ridge Detection of the Fuji ImageJ software.^28^ For the immuno-TEM, fibrils were applied to carbon-coated copper grids, blocked with 1% BSA and 0.1% Tween-20 in PBS, and incubated with primary antibodies, mouse antihuman αSyn monoclonal (SMC-532, StressMarq Biosciences Inc.) and rabbit antihuman Tau polyclonal (SPC-802, StressMarq Biosciences Inc.). After washing, the grids were treated with secondary antibodies, 6 nm colloidal gold-conjugated goat antimouse (115-195-166) and 12 nm colloidal gold-conjugated goat antirabbit (115-195-146, Jackson ImmunoResearch) at a 1:50 dilution in blocking buffer, washed again, and negatively stained with 2% uranyl acetate. The number of particles representing αSyn and Tau in each PFF is analyzed.

### 2.2 Cell Treatment and Seeding Activity

For seeding, PFFs were sonicated immediately after thawing from −80 °C using a water Bath-Cup Horn Sonicator, (Q125, Q-Sonica Newtown, CT, USA) at 70% amplitude in 1 s on/off cycles for 5 min. Cells were planted in triple wells in the 96-well plates (7500 cells/well) 24 h before the treatment and seeded with buffer (Opti-MEM), Lipofectamine reagent (Thermo Fisher Scientific Inc.), and various PFFs. We exposed cells to PFFs at a final concentration of 5 μg/mL, which, in the mixed conditions, contains 2.5 μg/mL each of Tau and αS. After treatment, cells were washed with PBS three times before the subsequent experiments. Images were taken from 15 fields per well with the Operetta CLS High Content Analysis System (PerkinElmer, Waltham, Massachusetts, USA) at each time point. Data obtained from three independent experiments were analyzed by the Harmony High-Content Imaging and Analysis Software (Figure S4B).

### 2.3 LDH-Cytotoxicity Assay

Cytotoxicity induced by different PFFs was tested with CyQUANT LDH Cytotoxicity Assay (Invitrogen, Massachusetts, USA). Briefly, cells were replenished with 100 μL fresh medium after 48 h of PFF treatment and grown in a cell culture incubator overnight. Subsequently, 50 μL of the medium was taken from each sample and combined with an equal volume of reaction mixture. 50 μL of stop solution was added after 30 min of incubation at room temperature protected from light. The untreated cells and cells treated with ultrapure water were determined as the maximum LDH activity and spontaneous LDH, respectively. The absorbance at 490 and 680 nm in triplicate wells was measured with a microplate reader and the final cytotoxicity was calculated as % cytotoxicity.

### 2.4 Immunofluorescence Staining

Cells grown on poly- *l* -lysine-coated coverslips were fixed with 4% paraformaldehyde in PBS. After permeabilization with 0.3% Triton X-100 in PBS for 20 min, cells were incubated with the blocking solution (5% bovine serum albumin/1% donkey serum in 0.1% Triton X-100/PBS). Cells were incubated with rabbit anti-p129-αSyn (Abcam #ab51253, 1:1000), and mouse anti-AT8-Tau (Millipore #AB144P 1:1000) diluted in blocking solution following fluorescent dye-conjugated secondary antibodies Cy3-donkey antimouse (1:800) and Cy5-antirabbit (1:600, Jackson ImmunoResearch). Nuclei were stained with DAPI (T3650, Invitrogen). Quadruple labeled images were captured with 63× magnified objectives using a Leica SP8 Scanning confocal microscope. Identical settings were loaded for laser power gain for each acquisition, and 30 fields from three independent experiments were analyzed. 3D remodeling based on Z-stack confocal images and colocalization analysis was conducted with IMARIS software (Oxford Instruments, UK). % colocalization and Pearson’s coefficients analysis were performed using the Coloc 2 plugin.

### 2.5 Cell Fraction and αSyn Solubility

Fractionation and lysis were performed as previously described.^29,30^ The pellets were first solubilized by sonication at 30% amplitude for 10 s in three cycles in 50 μL of 1% Triton X-100 lysis buffer (100 mM NaCl, 50 Mm Tris-HCl, 1 mM EGTA, 10 mM MgCl_2_), pH 7.2, protease (78430, Thermo Fisher Scientific) and phosphatase inhibitors (1862495, Thermo Fisher Scientific). After ultracentrifugation at 13,000 *g* for 20 min at 4 °C, the supernatant was collected as a Triton X-100-soluble fraction. The remaining pellets were collected and resolubilized in 50 μL of lysis buffer containing 1% sarkosyl on ice for 30 min and sonicated. The centrifuged supernatant was collected as a sarkosyl-soluble fraction. Proteinase K (PK) digestion was carried out as previously reported,^31^ and the sarkosyl-soluble fraction was incubated with PK (2 μg/mL) at 37 °C for 30 min. The digestion reaction was stopped by heating the samples to 100 °C for 10 min. Protein concentrations were measured with a BCA kit using standard procedures (Thermo Scientific, Pierce BCA protein kit, cat no #23225). Samples prepared for all the conditions were boiled in 4× Lamelli buffer and prepared for solubility testing via SDS-PAGE in Western blotting using anti-p129-αSyn (1:5000) and anti-αSyn (211) (Santa Cruz, #sc-12767, 1:5000).

### 2.6 Correlative Fluorescence and O-PTIR Imaging

The αSyn-GFP positive cells were initially imaged using a fluorescence microscope to identify the locations of the aggregates. Subsequent imaging was carried out using an O-PTIR mIRage Infrared Microscope (Photothermal Spectroscopy Corp., Santa Barbara, CA, USA) to collect individual cell or subcellular information for the aggregates inside the cells.

The detailed protocol for O-PTIR imaging is available in the Supporting Information. Finally, to eliminate spectral interference from bound fluorescent molecules, the intracellular amyloid aggregates with the repetitive arrangement of β-sheets were labeled with Amytracker 630 (1:500, Ebba Biotech) after all O-PTIR scanning. 8–10 cells from three independent experiments were scanned with 0.5–0.8 μm grid spacing to collect individual cells or subcellular information for the aggregates inside the cells.

### 2.7 Statistical Analysis

One-way ANOVA followed by Bonferroni’s post hoc comparison tests were performed in all statistical analyses. Multiple comparisons were conducted between different conditions. Statistics with a value of *P* < 0.05 were considered significant. Data analysis and plotting were performed using GraphPad Prism 9.5 and Origin 2022 software.

## 3 Results

### 3.1 Morphological Features of αSyn/Tau PFFs

In this step, we used AFM and immuno-TEM to analyze the morphology of αSyn monomers and three types of PFFs. We initially characterized the pure αS, Tau PFFs, and their copolymers using AFM as depicted in Figure S1A. The αSyn/Tau copolymers displayed a more compact, ribbon-like morphology compared to either αS or Tau fibrils alone. The average width diameter of the αS/Tau copolymers was significantly increased (αS&Tau3R: 5.62 ± 1.62; αS&Tau4R: 5.67 ± 1.89) compared to αS (4.54 ± 1.66 pixels) and Tau (Tau3R: 4.65 ± 1.79 pixels; Tau4R: 4.59 ± 1.65 pixels), as shown in Figure S1B. The histogram of fibril diameters provides a clear visual representation of their size distribution, indicating that the copolymers (∼5.5 to 6.0 pixels) are generally thicker than Tau (∼4.5 pixels) and αS (∼3.5 pixels) alone. Notably, the distribution of Tau3R and αS&Tau3R copolymers tends to follow a normal distribution more closely than those containing Tau4R (Figure S1C). Subsequent immunolabeling with antibodies specific to αS and Tau revealed that the copolymers contain both proteins. Interestingly, although no differences in thickness were detected between the two types of copolymers via AFM, the immuno-EM results revealed a distinct constituent proportion of αS and Tau in these two fibrils, with a ratio of approximately 1.06:1 (Tau: αS) in αS&Tau3R and 3.84:1 in αS&Tau4R (Figure 1A,B). These findings demonstrate morphological polymorphism of PFF used and more abundance of αSyn level in αS&Tau3R than αS&Tau4R.

**Figure 1:**
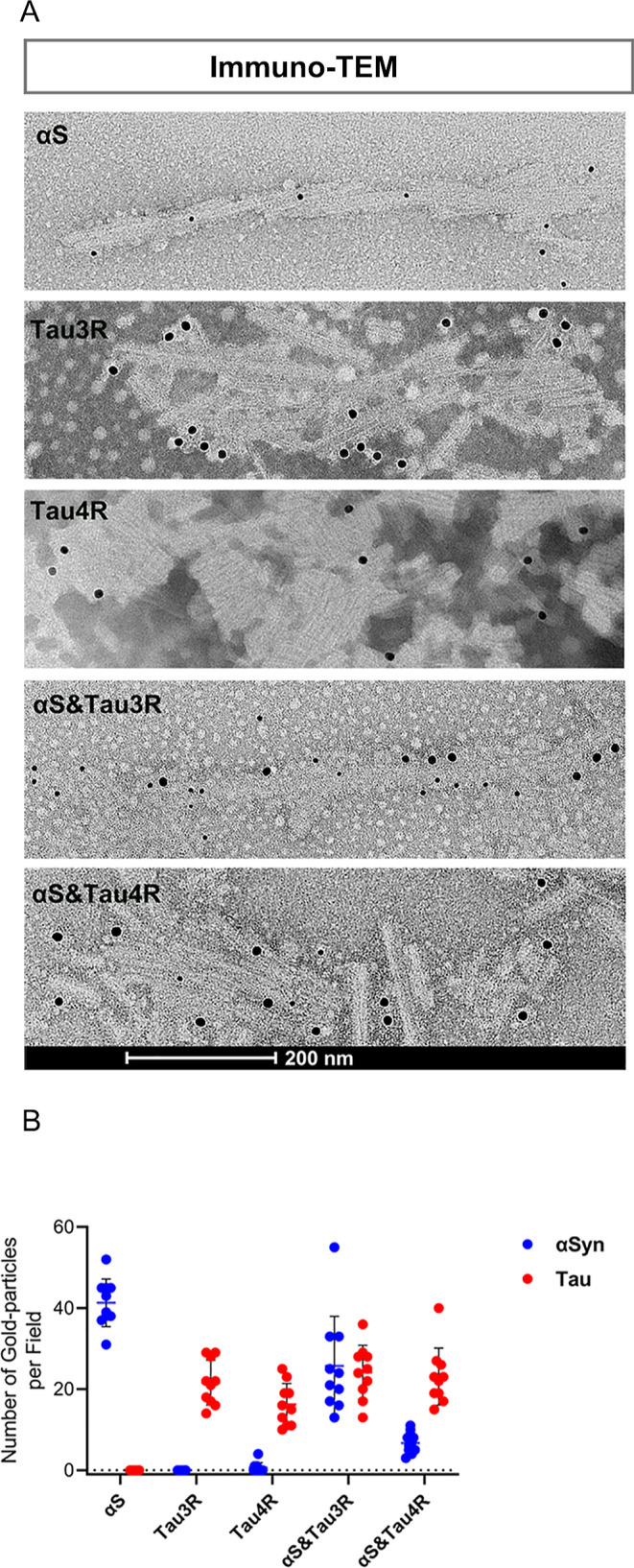
Morphological characterization of αS/Tau PFFs using immuno-EM. (A) Immuno-EM showing amyloid fibril samples, costained for αSyn and Tau. Double signals only appear together in the hybrid PFFs (αS&Tau3R and αS&Tau4R). Small size gold (6 nm): αSyn; Big-size gold (12 nm): Tau. Scale bar = 200 nm. (B) Numbers of αSyn and Tau positive particles for each fibril are collected from 10 individual fields and shown in the dot-plots.

### 3.2 Depiction of Amyloid Structure Signature of Pure PFFs with O-PTIR

Given the variable physical properties detected across different conditions, we hypothesized the existence of structural heterogeneity among different PFFs (Figure 2A). Using a commercial mIRage (Photothermal Corp., USA) O-PTIR microscope, we acquired spectra in the 1500–1750 cm^–1^ range (Figure S2A). The 1656 cm^–1^ absorbance has been associated with the protein backbone conformation and is specifically sensitive to the structures that form β-sheets, a shoulder centered around 1630 cm^–1^.^24^ Analyzing O-PTIR spectra, we found that mixed and hybrid PFFs displayed increased intensity of β-sheet compared to the pure αS seeds. In particular, both mixed and hybrid PFFs generated by αS and Tau3R displayed much greater levels of β-sheets compared to the same type of PFFs containing αS and Tau4R (Figures 2B and S2B). Interestingly, no elevation of intensity corresponding to β-sheets was observed in Tau PFFs and αS monomers. The O-PTIR spectra was first analyzed with the second derivatives method, which resolved more peaks associated with different structural components corresponding to amyloid fold (Figure 2C). Table S2 lists the specific bands detected. We found that both αS + Tau3R and αS&Tau3R PFF displayed more intensity at the band position 1630 cm^–1^ (β-sheets parallel) and 1640 cm^–1^ (random coils) than the corresponding types of PFFs formed with αS and Tau4R. Thus, αS&Tau3R exhibited the most abundant of both β-sheet structures across the conditions (Figure S2C, Table S3). Quantification of the different structural components depicted in Figure 2C is presented as a dot plot (Figure 2D).

**Figure 2:**
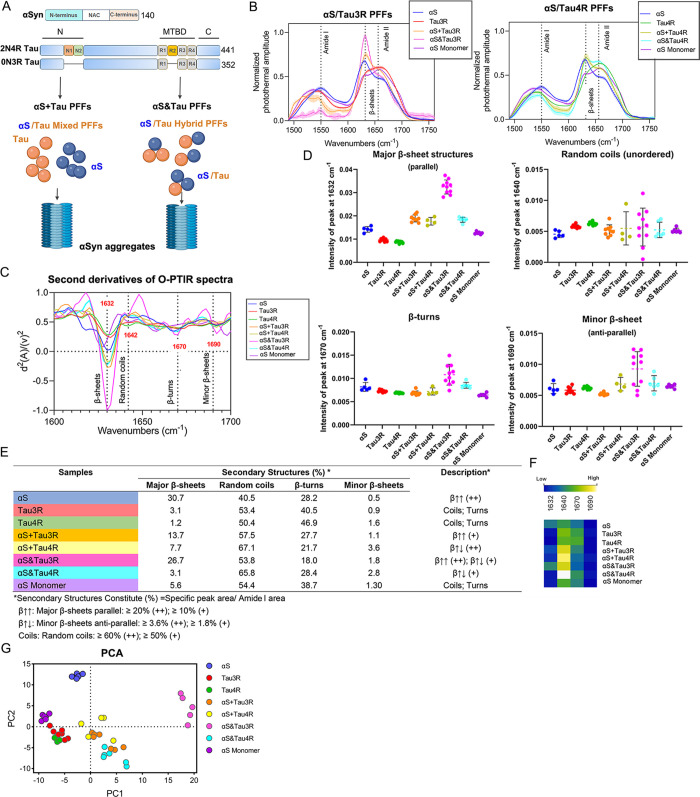
Distinct amyloid structures are presented in different αSyn/Tau PFFs. (A) Schematic illustration of seeding activities for αS, Tau isoforms 0N3R, 2N4R, and their mixed and hybrid PFFs (B) Averaged and normalized O-PTIR spectra for heteromeric PFFs, respectively, formed with αS/Tau3R and αS/Tau4R, compared to solely αS PFF and αS monomer. (C) Averaged and normalized second derivatives of the O-PTIR spectra (normalized to the maximum value across all conditions, with αS&Tau3R set as 1.0), with indicated peak position specific to amyloid structures. (D) The intensity of various amyloid structures in αS/Tau and their heteromeric PFFs with the second derivatives method, is shown in the dot plot (*n* = 5–8 samples/group). The significance of the multiple comparisons is provided in Data S3 (Supporting Information). (E) Acronym and secondary structures constitute different PFFs via peak fitting method. Amide I (1600–1700 cm^–1^) is resolved with a peak position specific to amyloid structures in (D). (F) Heatmaps of the original and average intensity of area under the curve in the O-PTIR spectra, corresponding to the amyloid band structures of each PFF sample. (G) The PCA of different PFFs are plotted with the first two principal components (PC1 and PC2).

Next, the secondary structure compositions of tested PFFs were modeled using a peak-fitting approach with the wavenumber of the bands identified by second derivatives (Figure S2D). To compare structures of different PFFs, we plotted peak areas corresponding to major structural components we observed in spectra such as β-turns, α-helices, and β-sheets.^32,33^ The proportion of peak areas in each PFF is summarized in Figure 2E, and the variations of PFF secondary structures are visualized by a heatmap (Figure 2F). Our findings reveal distinct secondary structure compositions among different PFFs, highlighting the structural specificity of αSyn/Tau interactions. Peak-fitting analysis of infrared spectra showed that (1) αS + Tau3R and αS&Tau3R PFFs exhibit a higher prevalence of parallel β-sheets (1630 cm^–1^); (2) αS + Tau4R and αS&Tau4R PFFs are characterized by a greater proportion of antiparallel β-sheets (1690 cm^–1^); (3) αS&Tau3R PFFs contain a mixture of parallel and antiparallel β-sheet structures, suggesting a more heterogeneous fibril composition; (4) Tau and αS monomers primarily consist of random coils and β-turns, confirming the absence of fibrillar structures in their native states. These findings underscore the unique structural fingerprints of each PFF type and suggest that αSyn/Tau interactions drive distinct fibrillar architectures, which may have implications for their biological properties.

To visualize structural differences, we employed a principal component analysis (PCA) plot to demonstrate the clustering and diversity of these PFFs (Figure 2G). Three principal components (PCs) successfully captured a significant variance, accounting for 90% of the total variance. Briefly, rare amyloids were observed from Tau PFFs and αS monomers (PC1^low^ and PC2^low^), in contrast to αS&Tau3R (PC1^high^ and PC2^high^). Similar distributions were reported for αS + Tau3R and αS + Tau4R (PC1^medium^ and PC2^medium^). The PCA plots, with wavenumbers of amyloid as the coordinates for the x and *y* axes, further illustrate the structural diversity of the eight PFFs (see Figure S3).

### 3.3 Seeding Activities of Various αSyn/Tau PFFs

We used HEK293 cells stably expressing human αSyn with A53T mutation tagged with eGFP (αSyn-GFP) to assess the seeding effects. The cells were treated with αSyn monomers and the other seven types of PFFs. Cells treated with 5 μg/mL BSA in phosphate-buffered saline (PBS) were used as the negative control (NC). Fluorescence images of all conditions were acquired at 6, 12, 24, and 48 h of incubation (Figures 3A and S4). We observed that pure αS, mixed, and hybrid PFFs induced the formation of αSyn inclusions (GFP positive accumulates) after 12 h, while no visible inclusions were found in cells treated with αS monomers or pure Tau PFFs. Interestingly, αS + Tau3R and αS&Tau3R exhibited a higher propensity to induce the formation of αSyn inclusions in cells. At 48 h, 15.5% of cells treated with αS + Tau3R and 14.9% treated with αS&Tau3R exhibited inclusions (Figure 3B). While the percentage of cells with inclusions remained relatively stable after 12 h, the inclusion size increased by 2–3 times for all of the conditions over 12–24 h. Among all groups, αS + Tau3R induced the largest area of intracellular αSyn inclusions (46.4 ± 11 pixels) (Figure 3C). Moreover, inclusions induced by αS&Tau3R showed the highest mean GFP intensity and highest compactness in this condition (Figure 3D). Additionally, we observed a decreased cell number at 48 h of incubation with αS + Tau3R (Figure 3E), suggesting potential cytotoxicity of the seeds. Using the lactate dehydrogenase (LDH) assay, we found that exposure to all conditions had minimal impact on cell viability, except for αS + Tau3R (5.8% cytotoxicity) (Figure 3F). In summary, these data revealed that αS + Tau3R and αS&Tau3R are most potent at inducing αSyn inclusions, however only inclusions induced by αS + Tau3R exhibited higher cytotoxicity as compared to other conditions. Thus, we demonstrated that different PFF polymorphs exhibit distinct biological properties, including varying effects on intracellular αSyn aggregation and local cytotoxicity.

**Figure 3:**
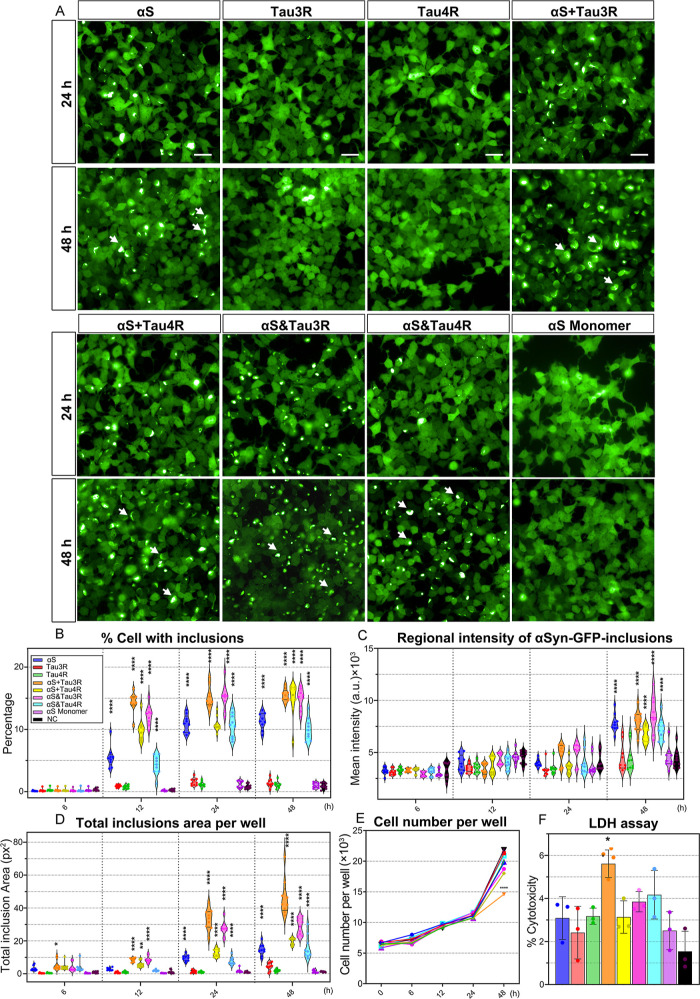
The seeding activity of αS/Tau PFFs on αSyn-A53T in GFP HEK cells. (A) The different seeding activities of various PFFs on the αSyn-A53T-GFP HEK cells at 24 and 48 h are shown in the fluorescent images (3 independent experiments/per group). Three characteristic inclusions per group are indicated with white arrows. Scale bars, 10 μm. (B–D) Quantifications of seeding effect as a function of time, the significance of the multiple comparisons is provided in Data S1 (Supporting Information) (E) Cell number per well recorded at each time point. (F) LDH assay of cells incubated with PFFs for 48 h, with a significance level set at *P* < 0.05.

### 3.4 Characterization of Intracellular αSyn Inclusions Seeded with Different PFFs

Next, we assessed the morphological, molecular, and biochemical properties of newly formed αSyn inclusions. Confocal image analysis revealed distinct morphological and temporal patterns of αSyn inclusions induced by different PFFs (Figure 4A). Thus, at 24 h, we observed the fast development of αSyn inclusions in the radial pattern with a compact core in cells treated with pure αS. We observed distinct morphologies of αSyn inclusion seeded by mixed and hybrid PFFs. Mixed PFFs generated reticular αSyn inclusions dispersed throughout the cytoplasm. These inclusions were characterized by tiny foci surrounded by lamellar inclusions, while the hybrid PFFs induced the formation of multiple solid and condensed inclusions. In addition, we examined the phosphorylation of αSyn and Tau, which is a prevalent pathogenic alteration for both proteins.^34,35^ Cells treated with different PFFs were labeled with p129 αSyn and AT8 Tau (Ser202/Thr205) antibodies. While p129 αSyn positive inclusions were identified after 12 h and increased over time, Tau phosphorylation was only detected after 24 h (Figures 4A and S5A,B). αSyn inclusions induced by αS + Tau3R and αS&Tau3R exhibited significantly greater phosphorylation of Tau and αSyn compared to other conditions, which was quantified by colocalization with Pearson’s correlation coefficient (Figures 4B and S5C,D).

**Figure 4:**
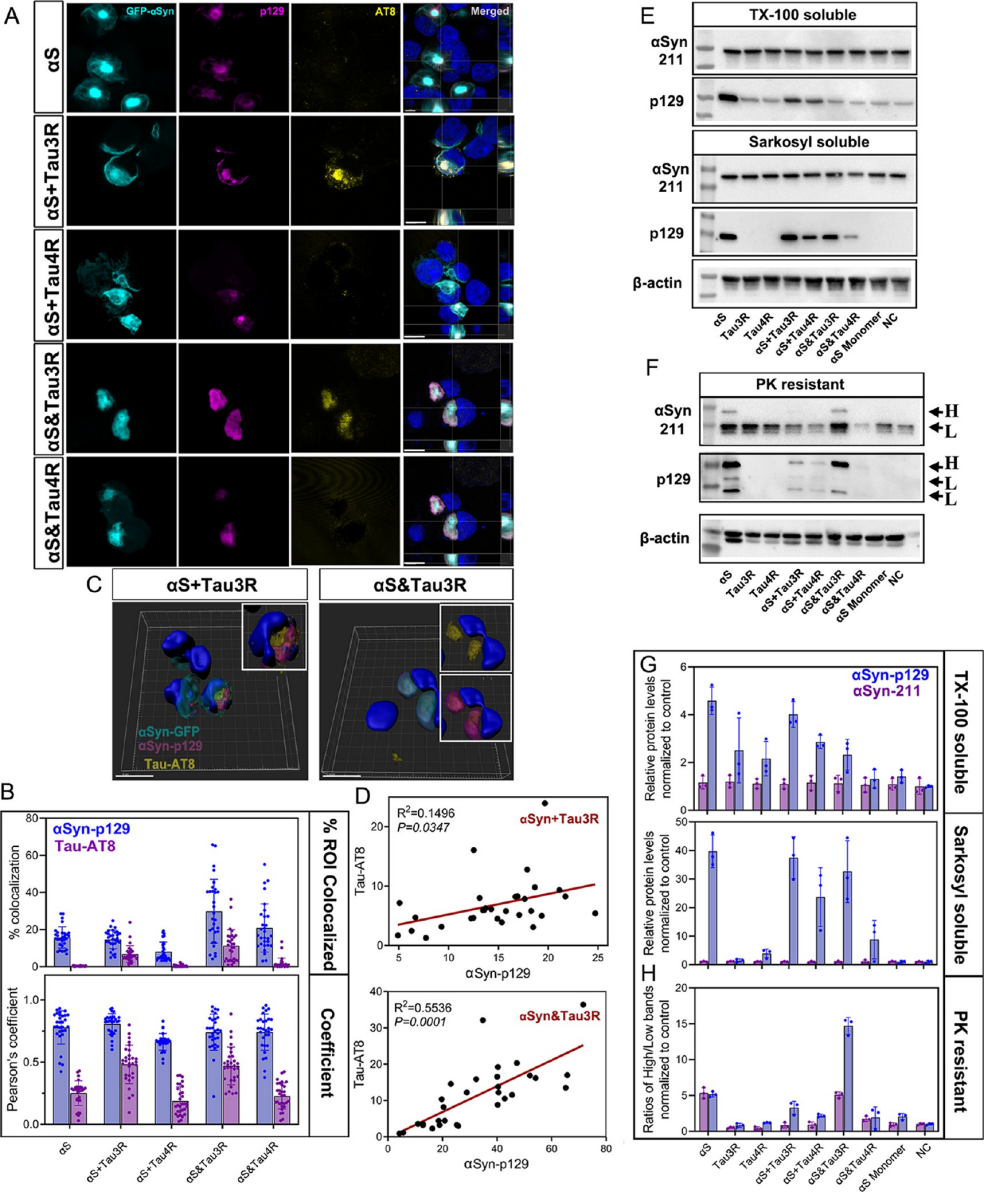
Intracellular αSyn inclusions induced by different αSyn/Tau PFFs exhibit distinct morphology and phosphorylation levels. (A) Confocal images of αSyn-A53T-GFP HEK cells at 48 h post seeding show distinct morphology and phosphorylation levels of αSyn (p-129) and Tau (AT8) in αSyn inclusions formed with different PFFs. Cells are counterstained with DAPI. Scale bars are 10 μm, (B) colocalization p-129 and AT8 with αSyn-GFP inclusions after 48 h are evaluated with % colocalization and Pearson’s coefficient. Bar plots are shown as mean ± s.d. (30 fields ∼ 100 cells/per group), the significance of the multiple comparisons over time is provided in Data S2 (Supporting Information), (C) 3D reconstruction of confocal micrographs showing different patterns of p-129 and AT8 colocalization in the αSyn inclusions induced by αS + Tau3R and αS&Tau3R, (D) linear correlations analysis for p-129 and AT8 in cells exposed to αS + Tau3R and αS&Tau3R, (E) distinct solubility αSyn inclusions are examined with SDS-PAGE and subsequent blotting with antibodies αSyn 211 and αSyn-p129. Cell lysates are extracted with 1% TX-100, 1% Sarkosyl, (F) cell lysates are digested in proteinase K (PK, 2 μg/mL). After PK digestion, the high (H: ∼40–45 kDa) and low (L: ∼35 kDa) molecular weights of αSyn can be detected, indicated with arrows, (G) quantification of αSyn solubility in 1% TX-100 and 1% Sarkosyl are shown in the bar plot as mean ± s.d, (H) quantification of αSyn resistant to 2% PK is shown in the bar plot as mean ± s.d.

To analyze the spatial distribution of phosphorylated αSyn and Tau, we performed 3D reconstructions and revealed two distinct patterns (Figure 4C): (i) αS + Tau3R led to more widespread Tau phosphorylation throughout the cytoplasm, with a stronger AT8 signal interweaving with p129 αSyn in the inclusions; (ii) in αS&Tau3R-seeded cells, AT8 predominantly merged with p129 αSyn within the inclusions. Using linear correlation analysis, we revealed that higher levels of αSyn phosphorylation were associated with phosphorylated Tau (Figure 4D). To further explore the properties of αSyn inclusions, we first assessed their solubility. We measured the levels of phosphorylated (p129) and total (pan-αSyn 211) αSyn in sequentially extracted fractions of cell homogenates, which were separated by electrophoresis and detected through Western blot (WB). Quantitative analysis showed that in homogenates of cells treated with αS and αS + Tau3R, higher levels of phosphorylated αSyn were observed in both the TX-100 and sarkosyl soluble fractions. In addition, αS + Tau3R and αS&Tau3R triggered higher αSyn phosphorylation than the corresponding types of PFFs containing αS and Tau4R (Figure 4E,G).

Next, we assessed the PK resistance of the αSyn inclusions. We found that PK digestion produced multiple low (L) molecular weight αSyn bands on the Western blots. We found that cells seeded with αS and αS&Tau3R demonstrated similar PK resistance. However, the ratio of high (H: ∼45 kDa) to low (L: ∼35 kDa) molecular weight αSyn bands was higher in the latter, implying a more complex conformational structure of the αS&Tau3R-induced inclusions (Figure 4F,H). Taken together, these data indicate that different PFF polymorphs influence αSyn seeding, affecting αSyn accumulation and the properties of αSyn inclusions, including phosphorylation levels and the PK resistance.

### 3.5 Correlative Fluorescence and O-PTIR Imaging Identifies Inherited Amyloid Characteristics from PFFs to Intracellular Aggregations

Our subsequent findings demonstrate that αSyn inclusions induced by different PFFs exhibit distinct structural heterogeneity, highlighting a strain-dependent conformational relationship between seeding PFFs and the resulting αSyn aggregates. Given the structural differences observed among various PFFs, we hypothesized that αSyn inclusions induced by different PFFs might exhibit structural heterogeneity. Using a correlative O-PTIR and confocal imaging approach, we identified variations in β-sheet distribution at submicron resolution, revealing that αSyn aggregates seeded by αS&Tau3R PFFs exhibited the highest levels of β-sheet structures. We observed that αSyn aggregates retained structural signatures of their respective PFF templates, with αS and αS + Tau4R favoring parallel and antiparallel β-sheets, respectively. In contrast, αS&Tau3R aggregates contained both β-sheet structures at high levels. Interestingly, cells treated with αS and hybrid PFFs also displayed elevated β-sheet content even in the absence of visible inclusions, suggesting that αSyn undergoes conformational modifications before forming detectable aggregates. Cells with and without inclusions were separately selected for analyses from each condition. Guided by GFP fluorescence, we used O-PTIR to examine the distributions of β-sheets (Figures 5A,B and S6A–D). Table S4 lists the peak ratios used in the second derivatives analysis of the cell. The ratio between 1628 cm^–1^ and 1656 cm^–1^ and between 1640 cm^–1^ and 1656 cm^–1^ (Figure 5C) are used to visualize the distribution of β-sheets. Figure 5D shows a ratio single energy map (1628/1656 cm^–1^) to display the distribution of major (parallel) β-sheet components in the cell selected in Figure 4B. The co-occurrence of different structures in a single cell is presented in the merged images (Figure 5E). Spectra analysis documented the presence of β-sheets that can be assigned to αSyn aggregation (Figure 5F). Cells with newly formed αSyn aggregates induced by αS, mixed, and hybrid PFFs had significantly higher levels of β-sheet structures (1628 cm^–1^) as compared to that of the control (NC) group. Interestingly, the treatment of cells with αS and hybrid PFFs also led to a notable elevation of β-sheets in cells without visible inclusions (Figure 5G). However, no β-sheet structures were detected in cells treated with αS monomers or Tau (Figure S6E). Finally, we performed multiple comparisons on the spectra extracted from cells with αSyn aggregates under all conditions (Figure 5H,I). We discovered that αSyn aggregates induced by αS&Tau3R, αS, and αS + Tau4R displayed comparable abundances of β-sheet structures. Notably, our findings indicated similar strain-dependent conformational structural characteristics in αSyn aggregates and their seeding PFFs shown in Figure 3E (Table S5). In particular, αS&Tau3R induced αSyn aggregates that displayed the highest level of both β-sheet structures. The aggregates seeded with αS and αS + Tau4R PFF are primarily featured by their high contents of parallel or antiparallel β-sheets, as observed in the original PFFs applied to cells. αSyn aggregates seeded with αS + Tau3R and αS&Tau4R, show moderately high parallel and antiparallel β-sheets, demonstrating similar features with the PFFs, respectively (Figure S6F, Table S5). The biochemical properties and secondary structures of aggregates seeded with different PFFs are summarized in Table 1.

**Figure 5:**
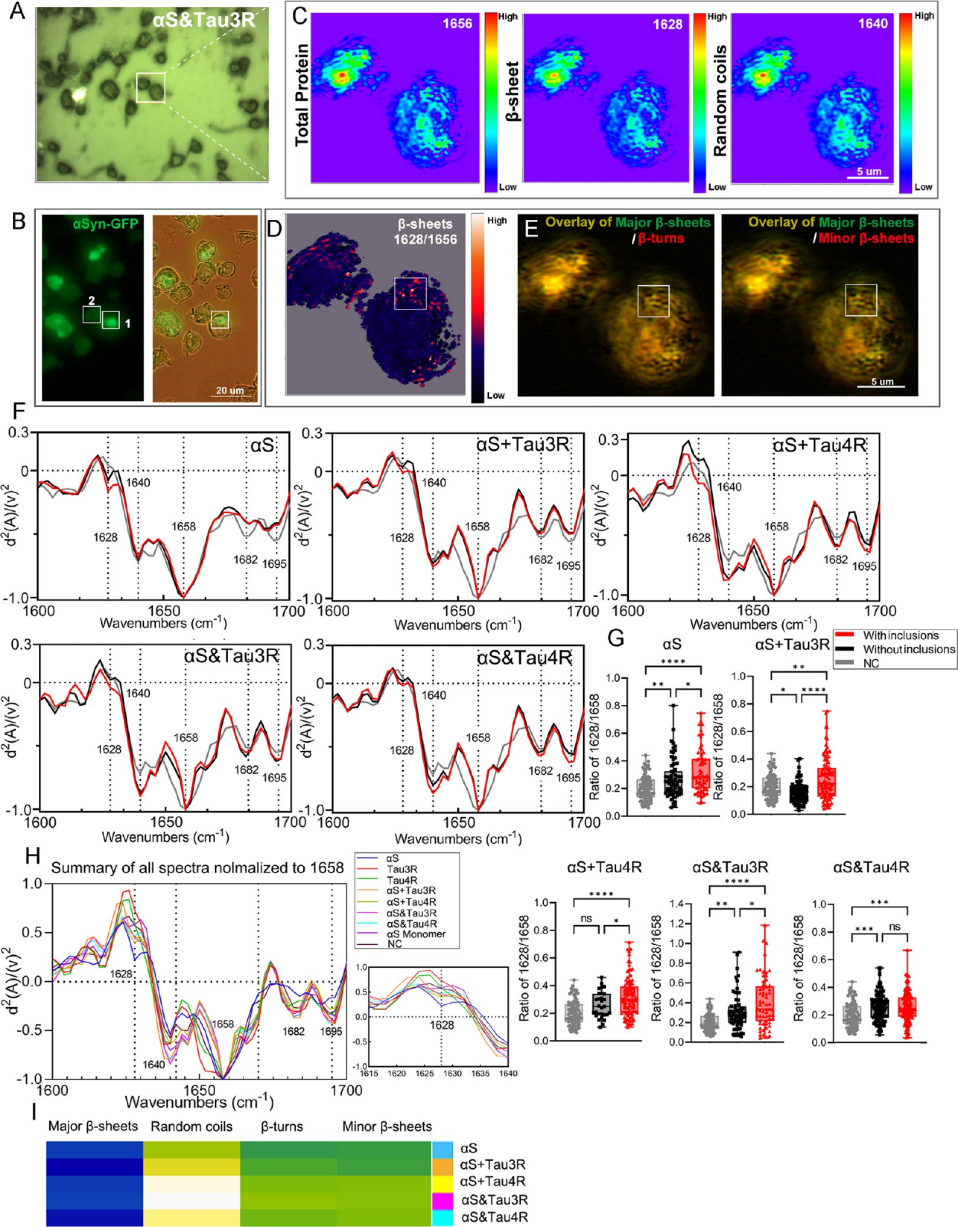
Correlative analysis of fluorescence and O-PTIR microspectroscopy in cells seeded with different PFFs. (A) Bright-field overview of cells grown on borosilicate glass grids used to identify the same object for fluorescence and O-PTIR imaging modalities. White insert indicates the cells imaged with fluorescence (C) and O-PTIR microspectroscopy (B). Scale bars are 20 μm, (B) single energy O-PTIR images at the specific wavenumbers corresponding to 1658 cm^–1^ (α-helix/total proteins); 1628 cm^–1^ (β-sheet major parallel); 1640 cm^–1^ (random coils). Scale bars are 5 μm, (C) fluorescence image overlap with bright field for the selected cells. White inserts indicate cells collected for amyloid structure analysis: 1: cell with inclusions and 2: cell without inclusions. The same object is marked in the O-PTIR ratio maps in (D,E). Scale bars are 20 μm, (D) O-PTIR ratio maps show the distribution of major β-sheet structures (1628 cm^–1^/1658 cm^–1^), overlay of the map with the masked visual image, (E) Overlays (yellow) of β-turns (1682 cm^–1^, red), minor β-sheet (1695 cm^–1^, red) with major β-sheet structures (green). Corresponding areas are indicated in (C,D) by white squares, (F) averaged and normalized second derivatives of O-PTIR spectra of cells with aggregates (red) and without aggregates (black) are compared the negative control (NC) groups (gray), (G) quantifications of major β-sheet structures intensity are shown in the bar plot as mean ± s.d. Spectra from five to seven cells in each group were pooled respectively for the statistical analysis, (H) averaged and normalized second derivatives of O-PTIR. A zoomed-in section of the β-sheet structure (1628 cm^–1^) is on the right of the main graph. No evident band for 1628 cm^–1^ was found for the cells seeded with Tau and αSyn Monomer, (I) heatmaps of the average intensity values of the second derivatives in the O-PTIR spectra, corresponding to the amyloid band structures of cells with inclusions in (H).

**Table 1:** Summary of the Biochemical Properties and Molecular Structures of Aggregates Seeded with Different PFFs^a^

PFFs			aggregates induced by PFFs					
groups	seeding potency	cytotoxicity	phosphorylation αSyn/Tau	solubility (αSyn-p129)	PK resistant	compactness	secondary structures	conformational change
αS	++		+/—	+++	++	++	β↑↑ (++); β↑↓ (+); C (+); T (+)	+
Tau3R			—/—	+				
Tau4R			—/—	+				
αS + Tau3R	+++	+	+/+	+++	+	+	β↑↑ (+); β↑↓ (+); C (++); T (+)	
αS + Tau4R	+++		—/—	++	–	+	β↑↑ (++); β↑↓ (++); C (+++); T (++)	
αS&Tau3R	+++		++/++	++	+++	+++	β↑↑ (+++); β↑↓ (+++); C (+++); T (+++)	++
αS&Tau4R	+		++/—	+	+	+++	β↑↑ (+); β↑↓ (++); C (+++); T (++)	+
αS monomer				+				

a Seeding potency: % cell with inclusions ≥ 15% (+++); ≥ 10% (++); ≥ 5% (+) cytotoxicity: % cytotoxicity ≥ 5% (+). phosphorylation αSyn/Tau: % cells positively labeled with aSyn-p129 ≥ 20% (++); ≥ 10% (+)/Tau-AT8 ≥ 5% (+); ≥ 10% (++). Solubility (αSyn-p129): TX-100 soluble relative level normalized to control ≥ 4% (+++); ≥ 2% (++); < 2% (+). PK resistant (αSyn-p129): relative level normalized to control ≥ 10% (+++); ≥ 5% (++); ≥ 3% (+); < 3% (-). Secondary Structures (ratio normalized to the total protein): major β-sheets: β↑↑ ≥ 0.35 (+++); β↑↑ ≥ 0.3 (++); β↑↑ ≥ 0.2 (+); minor β-sheets: β↑↓ ≥ 0.7 (+++); β↑↑ ≥ 0.6 (++); β↑↑ ≥ 0.5 (+); random coils: C ≥ 0.9 (+++); C ≥ 0.8 (++); C ≥ 0.7 (+); β-turns: T ≥ 0.7 (+++); T ≥ 0.6 (++); T ≥ 0.5. Conformational changes: shifted β-sheets observed in aggregates ≥ 2 segments (++); ≥ 1 segment (+).

This data implies that αSyn undergoes conformational modifications before forming visible aggregates and that strain-distinct aggregations inherited the amyloid characteristics from their templates. These findings indicate that αSyn aggregation follows a strain-specific pathway, inheriting amyloid characteristics from the seeding PFFs, which may have important implications for understanding pathogenic mechanisms in synucleinopathies and tauopathies.

### 3.6 Amyloid Dye-Guided O-PTIR Reveals Structural Evolution of Intracellular αSyn

For a more detailed structural and spatial characterization of intracellular αSyn aggregates induced by different PFFs, we integrated O-PTIR with fluorescence imaging using Amytracker (Ebba Biotech AB, Sweden), a dye sensitive to β-sheet structures.^36^ This approach allowed us to track the progressive fibrillization of αSyn aggregates over time, revealing the formation of mature amyloid structures after 60 h.

Assessing the colocalization of GFP and Amytracker, we detected that the fibrillization of αSyn aggregates induced by PFFs begins after 24 h and grows over time (Figure S7). Therefore, the incubation period was extended to 60 h to ensure that cells treated with PFFs contained mature amyloid aggregates. Each αSyn aggregate was segmented into “GFP-Core”, “GFP-High”, and “GFP-Low” regions based on the overlap of Amytracker and αSyn-GFP. αSyn aggregates induced by αS&Tau4R were divided into “GFP-Core” and “GFP-Low” regions following the criteria above, as indicated in Figure 6A. It appeared that the compact “GFP-Core” of the αSyn aggregates possessed the highest intensity of total β-sheets compared to “GFP-High” and “GFP-Low” regions in most of the conditions (Table S6). Counterintuitively, we found no significant differences between the subregions of the aggregates seeded with mixed PFFs (Figure 6B,C). After 60 h, aggregates formed with αS developed more β-sheets than those formed with αS&Tau3R and αS + Tau4R. The “GFP-Core” of αS-seeded aggregates possessed the highest major β-sheet structure content, followed by “GFP-Core” and “GFP-High” regions of the aggregates formed by αS&Tau3R. Notably, a shifted band of β-sheet, from 1628 cm^–1^ to 1632 cm^–1^, was observed across the areas mentioned above, suggesting a β-sheet conformational change occurred under the conditions.^24^ All subregions of αSyn aggregates induced by αS + Tau3R exhibited the lowest amyloid content, consistent with individual cell data at 48 h (Table S5). In contrast, aggregates generated by αS + Tau4R displayed a higher presence of random coils (Figure 6D,E). Table S6 summarizes the intensity ratio of secondary structures throughout αSyn aggregate segments.

**Figure 6:**
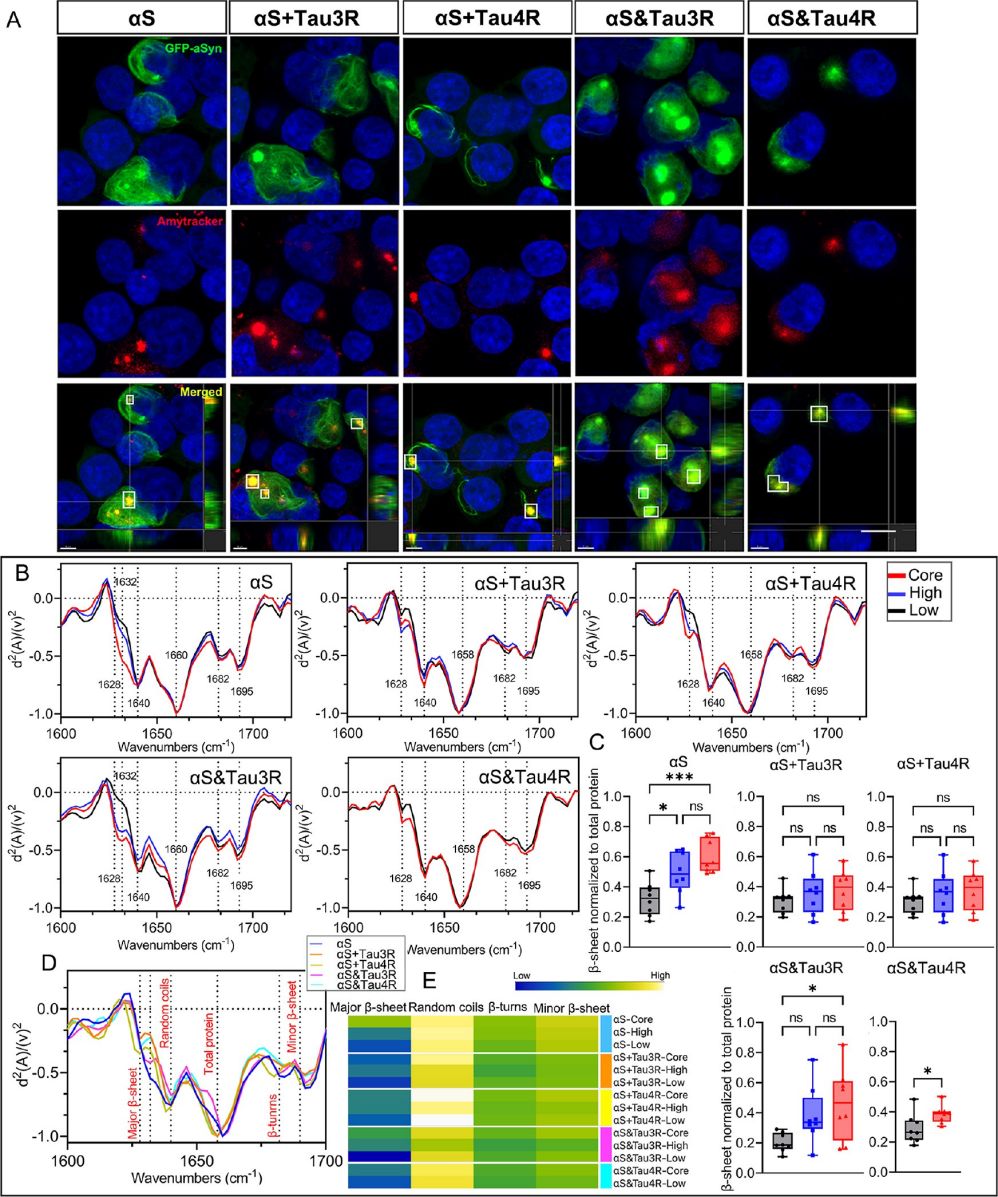
O-PTIR imaging guided by amyloid dyes depicting the subcellular distribution of amyloid structures in αSyn-GFP aggregate. (A) Confocal images for mature amyloids of the aggregates are labeled with Amytracker (red) and overlay with strain distinct αSyn-GFP aggregates (green) seeded from different PFFs at 60 h. Single intracellular aggregate is classified into core, high and low aggregate segments according to the overlapping of Amytracker and GFP. GFP-Core: (Amy+ and GFP+) in aggregates; GFP-High: (Amy– and GFP+) in aggregates; GFP-Low: No visible Amytracker and GFP labeling. White squares demonstrate the core regions of aggregates selected in each group. The compact aggregates seeded with αS&Tau4R can be classified into only core and low areas based on the above standards. Scale bars are 10 μm, (B) averaged and normalized second derivatives of O-PTIR spectra taken from the counterparts of the strain distinct αSyn-GFP aggregates. Shifted band of β-sheet structures from 1628 cm^–1^ to 1632 cm^–1^ is observed in the core of the αSyn seeded aggregates, and in the core and high of aggregates seeded with αS&Tau3R, (C) quantifications of the intensity of the major β-sheet structures are shown in the bar plot as mean ± s.d. Spectra from eight cells with typical aggregates in each group were included for statistical analysis, (D) averaged and normalized second derivatives of O-PTIR spectra of different subcellular parts of aggregates in strain distinct αSyn-GFP aggregates seeded with various PFFs, (E) heatmaps of the average intensity values of the second derivatives in the O-PTIR spectra of different subcellular parts of aggregates in strain distinct αSyn-GFP aggregates, corresponding to the amyloid band structures of cells with aggregates in (D). Intensity values are marked on the heatmap.

Finally, the total β-sheet (1632 cm^–1^) distribution was visualized in 3D density images, with the height and color intensity representing the content of β-sheets (Figure 7A–C). Our analyses revealed that the highest total β-sheets are generally found within the “Core” of aggregates, consistent with the spectral results (Figure 7D). However, when comparing the hyperspectral image after normalization to total protein (1658 cm^–1^), we found that the subcellular distribution of β-sheets demonstrated distinctive patterns for the newly formed αSyn aggregates induced by different PFFs. In the aggregates induced by αS and mixed PFFs, the highest normalized β-sheets were found in the periphery rather than the center of the “Core”. In the aggregates induced by the hybrid PFFs, the “Core” part displayed both higher total β-sheets and normalized β-sheets. This phenomenon was particularly pronounced in multiple foci of αSyn aggregates induced by αS&Tau3R (Figure 7D). Intriguingly, the regions with the highest normalized β-sheet content, corresponded to the initial formation sites of the αSyn aggregates induced by the mixed and hybrid PFF, as observed in the fluorescent images of αSyn-GFP at different time points (Figure S7). This result indicates an accumulation of αSyn concurrent with its fibrillization. The subcellular distribution pattern and intensity of β-sheets reflect distinct processes of αSyn aggregation induced by mixed and hybrid PFFs, highlighting their distinct origins and evolutionary courses.

**Figure 7:**
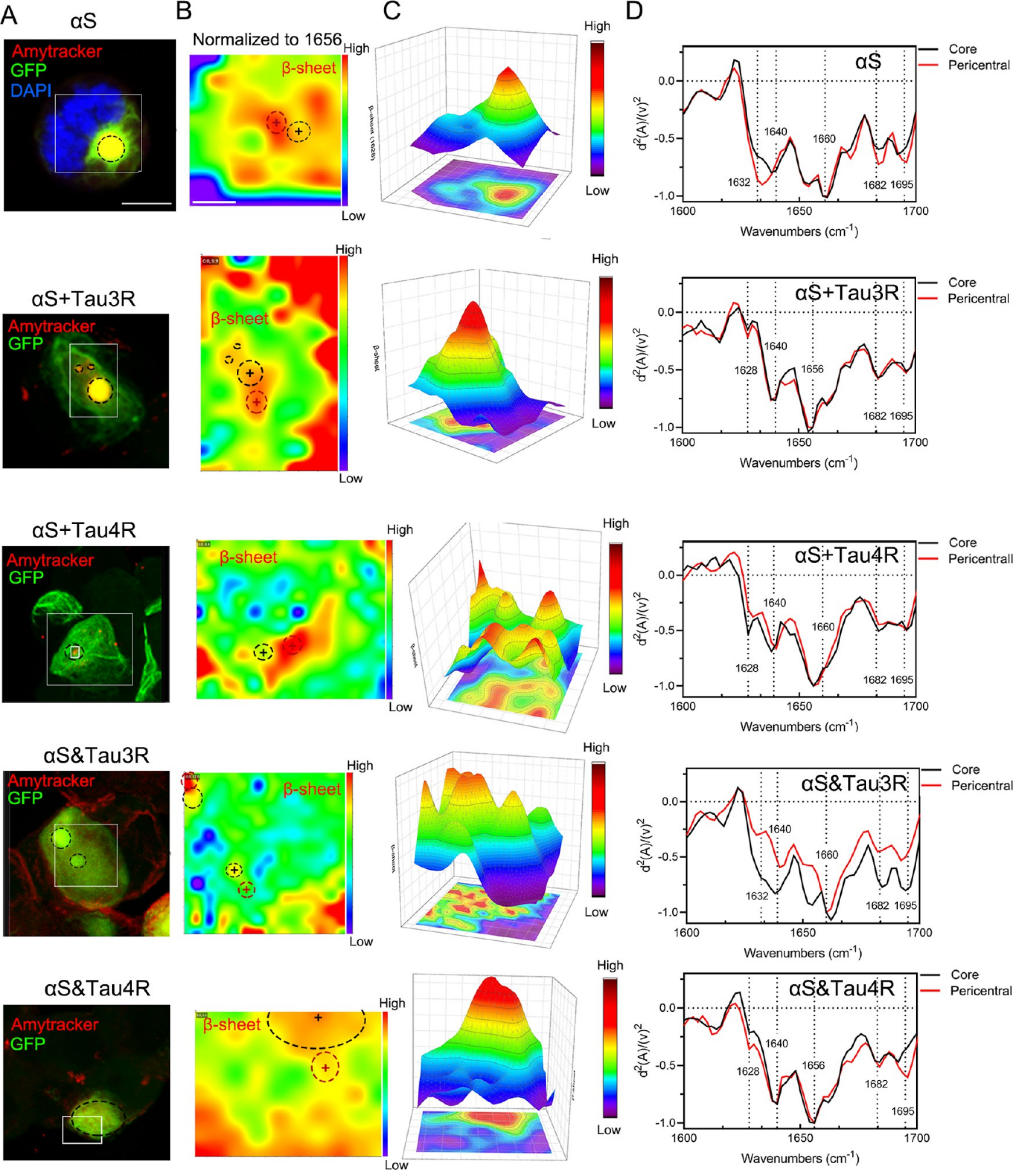
O-PTIR hyperspectral map distinguishes different patterns of β-sheet subcellular distributions in αSyn-GFP aggregates. (A) Overlayed confocal images of cells with aggregates seeded with different PFFs, Amytracker (red), GFP-αSyn aggregates (green). GFP-Core is indicated by a circle outlined with a black dashed line. White insert indicates the parts shown in the hyperspectral map. Scale bars are 5 μm, (B) hyperspectral map of β-sheets (1632 cm^–1^) normalized to total protein (1658 cm^–1^). The intensity of the rainbow represents level of β-sheets. The identical core region in A is marked on the hyperspectral map. The vicinity with high β-sheets (pericentral region) selected is indicated by the circle outlined with a red dashed line. Hyperspectral map of O-PTIR reveals different β-sheet subcellular distribution patterns of the aggregates. Scale bars are 2.5 μm, (C) 3D density plot with projection demonstrates the subcellular distribution of total β-sheets (1632 cm^–1^) in an individual cell with aggerate. The intensity of the rainbow represents levels of β-sheets, (D) averaged and normalized second derivatives of O-PTIR spectra from two different subcellular parts of aggregates in the circle (B) reveal different β-sheet subcellular distribution patterns of the aggregates. The crosses indicate the spot which corresponds to the spectra.

Thus, by integrating hyperspectral and fluorescence imaging, we mapped the subcellular distribution of β-sheets and identified distinct structural patterns of newly formed αSyn inclusions induced by different PFF seeds. By capturing these molecular-level changes in their native cellular environment at submicron resolution, our analyses revealed that β-sheet accumulation and structural rearrangements evolve over time, highlighting the spatial progression of αSyn aggregation.

## 4 Discussion

This study investigated αSyn and Tau structural perturbations using cell biological techniques and O-PTIR. We identified distinct physiological and structural properties of intercellular αSyn inclusions induced by pure, mixed, and hybrid PFFs. Notably, αS&Tau3R seed exhibited the highest amyloid content and seeding potency, whereas Tau alone failed to seed αSyn inclusions. Both αS + Tau3R and αS&Tau3R showed higher β-sheet contents and increased phosphorylated αSyn and Tau levels compared to their Tau4R counterparts, though they formed structurally distinct inclusions. Fluorescence-guided O-PTIR imaging revealed that aggregates retained the amyloid characteristics of their seeding PFFs, while spectral analysis highlighted strain-specific similarities and differences. Additionally, amyloid dye-guided O-PTIR imaging provided a detailed subcellular distribution of fibrils, reinforcing the distinct structural patterns induced by different PFF polymorphs.

### 4.1 β-Sheet Propensity within the Templates Affects αSyn and Tau Cross-Seeding

Previous studies have proven that αSyn directly interacts with the microtubule-binding domain (MTB) of Tau through its C-terminus, undergoing conformational change and synergistically promoting amyloid fibrillation.^15^ An early coarse-grained aggregate model indicated that the kinetic routes and morphologies of seeded peptide aggregation are controlled by β-sheet propensity.^37^ Furthermore, DLB brain extracts exhibited a greater inclination to initiate pathological protein aggregation than PD, impacted mainly by rod-shaped fibrils containing β-sheets.^38^ As such, in the present study, we initially compared the different secondary structures of various αSyn and Tau fibrils using O-PTIR and explored whether their structural diversity affects αSyn and Tau cross-seeding. Our findings revealed that pure Tau contains fewer β-sheets compared to αSyn fibrils, aligning with a recent report that demonstrated Tau fibrils exhibit a dynamic and unstable β-sheet arrangement in the early stages, as uncovered by nanoscale infrared spectroscopy and synchrotron X-ray diffraction.^39^ Contrasting with previous studies where αSyn seeding induced Tau aggregation,^13,14^ we found that Tau fibrils with lower β-sheet content scarcely induce the formation of intracellular αSyn inclusions. This is consistent with the in vivo studies in which αSyn fibrils caused modest levels of phosphorylated αSyn and Tau pathology in a tauopathy mouse model (PS19). In contrast, Tau fibrils had limited effects on αSyn aggregation in synucleinopathy mouse models (M20).^40^ Previous studies have shown that the non-amyloid-β component (NAC) core of αSyn (residues 61–95) can serve as an inducer of Tau polymerization, emphasizing the crucial role of β-sheets in their cross-seeding.^5,41^ The current study found that mixed and hybrid αSyn/Tau PFFs generated more secondary structures and displayed higher seeding potency than pure αS. Our observations align with previous reports, demonstrating that Tau/αSyn copolymers exhibited a distinct conformation and increased PK resistance, resulting in increased aggregation in αSyn A53T transgenic mice.^10^ Interestingly, we found that the mixed and hybrid PFFs made from αS and Tau3R showed higher β-sheet composition, stronger seeding activities, and induced more robust αSyn and Tau phosphorylation compared to their counterparts containing αS and Tau4R. Notably, αS&Tau3R initially displayed the most abundant β-sheets and seeding activities, whereas αS&Tau4R showed the least. Our immune-EM results further elucidated this discrepancy, which determined a higher proportion of αSyn in αS&Tau3R than in αS&Tau4R, despite both being synthesized using the same method. This divergence may be caused by structural variations in MTB repeat domains of two Tau 3R isoforms, for instance, hexapeptide motif VQIINK,^42^ which functions as the basis for Tau dimerization only present in Tau 4R. Furthermore, previous studies proved that the three-repeat interacts directly with the highly negatively charged C-terminus of αSyn, in contrast to the four-repeat, which interacts with both highly negatively charged C-terminus and the hydrophobic peptide in the NAC region of αSyn.^6,43–46^ In summary, our data suggests that the β-sheet propensity within the templates plays a critical role in determining the distinct cross-seeding activities of αSyn and Tau. These activities are significantly influenced by the specific Tau isoforms, with Tau3R having a stronger mediation effect compared to Tau4R.

### 4.2 Strain-Distinct Aggregates Inherit Amyloid Characteristics of the Seeds

Different αSyn strains display unique seeding patterns and pathological effects, profoundly influencing the progression and heterogeneity of synucleinopathies.^3,38,47^ Emerging evidence indicates that the distribution pattern of LBs in AD patients differs dramatically from that of PD patients, with LBs identified mainly in the limbic and olfactory regions but less in the substantia nigra and brainstem.^48–51^ These findings further support the idea that different αSyn strains might be involved in the co-occurrence of Tau and αSyn pathology. Using fluorescence-guided O-PTIR, we discovered distinct conformational structures in the αSyn aggregates induced by different PFFs. PFFs with either higher major or minor β-sheets (αS, αS&Tau3R, and αS + Tau4R ) typically lead to abundant amyloids in the aggregates, implying both components are crucial and may synergistically impact the secondary structure formation. Notably, we found these strain-specific aggregates inherit the amyloid characteristics of the templating seeds. The conformational memory effects observed here are in line with previous findings, indicating that structural alterations could be transmitted by seeding during in vitro and in vivo amplification.^52,53^ αSyn strains derived from DLB, MSA, and PD were able to preserve their specific structural and morphological features in the fibrils generated with real-time quaking-induced conversion (RT-QuIC) and protein misfolding cyclic amplification (PMCA), allowing for distinguishing distinct synucleinopathies.^2,38,54^ Moreover, systemic and local delivery of αSyn oligomers, ribbons, and fibrils triggered the production of new fibrils that maintain their original structural properties as they propagate.^55^ Interestingly, the strain-specific features in aggregates gradually diminished in the late stage (60 h), with αS-seeded aggregates exhibiting the highest β-sheets across all groups. Our results are consistent with the in vivo data, showing that injecting αSyn/Tau copolymers was less effective than αS alone in inducing αSyn pathology in mouse models of synucleinopathies.^40^ Previous research indicates that environmental factors, including temperature, pH, and cellular milieu, significantly impact the secondary nucleation of αSyn.^53,56,57^ Subsequent research demonstrated that sequence similarity and conformational compatibility between the seeds and the monomer control the elongation effectiveness of heterologous aggregation of αSyn.^58,59^ This finding may explain the abundant β-sheets and strong pathogenicity observed in strains induced by αS alone. Moreover, we identified a significant β-sheet peak spectral shift in aggregates seeded with αS and αS&Tau3R, which correlates with enhanced PK resistance. Similar shifted bands for unordered β-sheets have been detected with O-PTIR in AD transgenic neurites in our previous study,^60^ revealing the polymorphic nature of amyloid structures. Thus, the results suggest that these two strains possess unique β-sheet conformational forms compared to the others. We propose that the polymorphism may be attributed to post-translational modification and their additional contacts with other molecules, such as nucleic acids, lipids, and metal ions, as evidenced by prior structural studies using EM and cryo-EM.^11^

### 4.3 Distinct Patterns and Molecular Mechanisms for αSyn Aggregation Seeded with Mixed and Hybrid PFFs

Several studies have proven the copathology between Tau and αSyn.^6,7,10,61^ However, the detailed mechanism of their cross-seeding is still unclear. Based on related studies, we proposed three models for the molecular mechanisms recently:^62^ (1) Tau and αSyn directly bind to each other and form coaggregates; (2) Tau and αSyn aggregate independently in the same cell; and (3) Tau and αSyn serve as the template of the other protein. With three types of PFFs, the current study has largely validated the feasibility of these mechanisms in αSyn aggregation, except for templating of αSyn by the pure Tau. Notably, the data with O-PTIR supports two distinct aggregation patterns triggered by hybrid and mixed seeds identified in conventional cell chemistry, which match models 1 and 2 (Figure 8). Specifically, aggregates generated by αS&Tau3R show substantial overlap between phosphorylated Tau and αSyn, which correlates to the most abundant amyloid formations. In contrast, inclusions formed by αS + Tau3R displayed minimal amyloid content and minor overlaps within the aggregates, with extensive tau phosphorylation outside. Our findings deepened our understanding of the disputed outcomes reported in previous in vivo studies involving two different types of seeds. In mouse models transduced with AD lysate-derived tau and αSyn fibrils mixture, the absence of endogenous mouse αSyn reduces the accumulation and spreading of Tau. At the same time, the lack of Tau does not affect the seeding or spreading capacity of αSyn.^63^ However, in mice injected with Tau-modified αSyn or αSyn/Tau copolymers, knockout of Tau attenuated αSyn propagation and Tau-associated mitochondrial toxicity.^10^ The combined results highlight the different roles of Tau in their coaggregative and cooperative actions. While the hybrid αSyn/Tau seeds tend to recruit monomers to form aggregates integrally, in the αSyn/Tau mixture, only a minor fraction of Tau facilitates aggregation, suggesting limited amyloid formation was primarily driven by αSyn. O-PTIR proved the presence of these two distinct patterns. Previously, we reported a notable elevation of β-sheets in focal Aβ aggregates at synaptic terminals preceding the formation of amyloid plaque in AD mice.^24^ Here, we observed a similar phenomenon in cells without apparent aggregates seeded with αS and hybrid but not mixed PFFs. This implies that different early conformational alterations occur during the two types of aggregation. Moreover, the high-resolution hyperspectral image demonstrated that the highest normalized β-sheets were distributed in the core and peri-core regions of hybrid and mixed seed aggregates, respectively, corresponding to the initial formation sites of the inclusions. Growing evidence suggests αSyn intermediate oligomeric and proto-fibrillar species also contain β-sheet and helical conformations.^64,65^ The morphological and mechanical properties indicate an increase in β-sheet content as the structures progress from oligomers to mature fibrils.^66–68^ Therefore, our findings imply different seeding and elongation processes and conformational evolutions between the two models, indicating their different molecular mechanisms.

**Figure 8:**
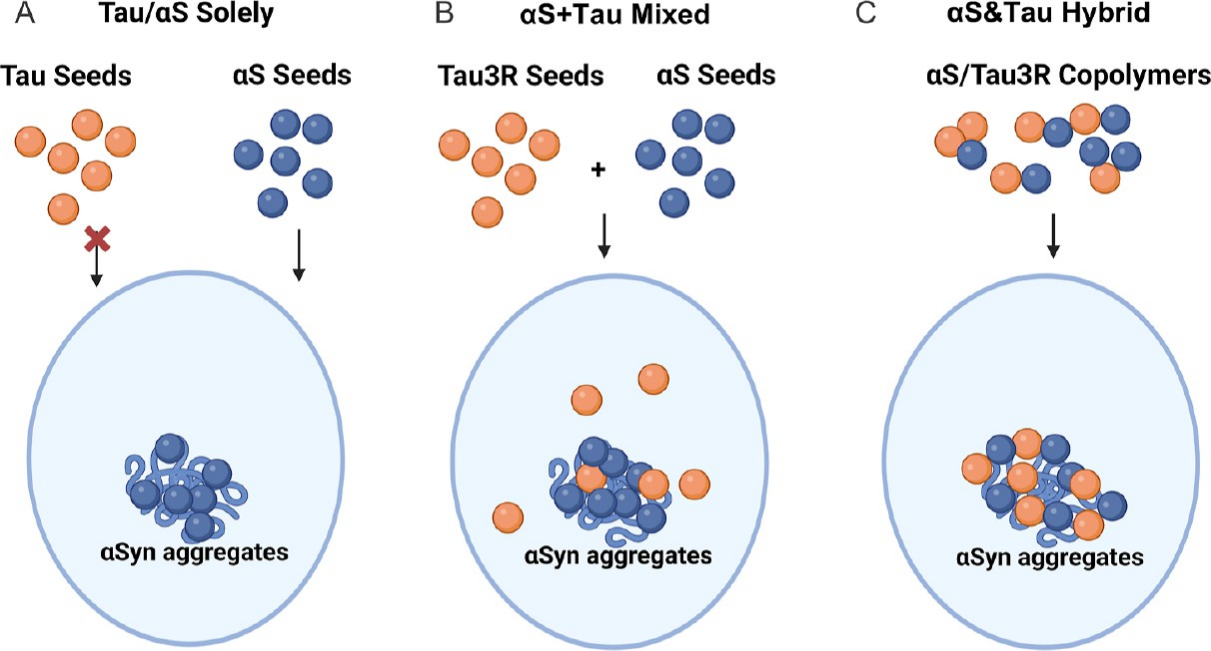
Mechanisms of different types of αSyn/Tau PFFs cross-seeding αSyn aggregates presumed in the current study. (A) Pure αS seeds, but not solely Tau seeds, can serve as the template and initiate αSyn aggregates. (B) In mixed αS + Tau3R seeds, αS and Tau3R act in different roles in the formation of αSyn aggregates. αS seeds serve as the template and initiate αSyn aggregates. Only a small proportion of Tau3R seeds template or facilitate αSyn aggregates, while the others trigger a widespread intracellular Tau phosphorylation. The cross-seeding process leads to the lowest content of amyloid structures in the aggregates. (C) In hybrid αS&Tau3R seeds, αS&Tau3R copolymers act dependently to recruit αSyn and Tau monomers and form αSyn and Tau aggregates. The cross-seeding process leads to the highest content of amyloid structures in the aggregates.

Recent studies have applied O-PTIR imaging to AD patient brain tissue^69^ and transgenic animal models, including tau transgenic mice (rTg4510),^70^ P301S mice, and 5xFAD mice.^71^ Using O-PTIR, Wang et al.^71^ spatially resolved β-sheet-rich amyloid structures in both 5xFAD and P301S mice and correlated them with lipid composition, suggesting that lipids may differentially influence Aβ and Tau aggregation. These and other studies^25,27^ have primarily focused on characterizing individual amyloids within their native tissue environments. While such tissue-based analyses provide valuable insights into the structural organization of amyloids, the inherent complexity of brain samples—comprising diverse cell types and extracellular matrix components proposes significant challenges for mechanistic interpretation in vivo. To address these challenges in a more controlled and tractable system, we employed a cellular model in the present study to investigate structural differences not only induced by individual amyloid seeds (e.g., αS or Tau) but also by mixed or hybrid αS/Tau seeds. The major advantage of using a comparatively “simpler and more homogeneous” cell model is the ability to tightly control experimental variables and directly study protein aggregation dynamics and β-sheet enrichment under defined conditions. Using this model, we demonstrated that distinct amyloid seeds generate unique intracellular β-sheet-rich aggregation patterns, and that de novo aggregates can inherit the conformational features of their corresponding in vitro PFF. Our work allowed us to discriminate the early, seed-specific structural transitions, underscoring the translational relevance of the correlative O-PTIR/confocal imaging approach in deciphering amyloid strain biology.

One limitation of our study is the use of endogenous GFP-tagged αSyn, which may potentially introduce artifacts by influence on its aggregation behavior and structural properties.^72^ However, since all experiments used the same GFP construct, the comparative analyses remain valid for assessing the relative differences in fibril behavior under controlled conditions. Furthermore, considering that all PFFs used in this study were recombinant, further study would be important to validate these findings in post-mortem samples from different diseases, for instance, AD with αSyn pathology, DLB, and PD with Tau pathology, to understand the clinical relevance of our findings. This could lead to better disease definitions based on understanding underlying biology and better clinical trials inclusion/exclusion criteria based on specific drug interventions.

## 5 Conclusion

With cell biology techniques and O-PTIR submicron imaging, the current study identified distinct physiological and structural properties of αSyn inclusions induced by pure, mixed, and hybrid PFFs. Notably, αS&Tau3R exhibited the highest amyloid content and seeding potency, whereas Tau alone failed to seed αSyn inclusions. Both αS + Tau3R and αS&Tau3R showed higher β-sheet content and increased phosphorylated αSyn and Tau levels compared to their Tau4R counterparts, though they formed structurally distinct inclusions. Fluorescence-guided O-PTIR imaging revealed that aggregates retained the amyloid characteristics of their seeding PFFs, while spectral analysis highlighted strain-specific similarities and differences. Additionally, amyloid dye-guided O-PTIR imaging provided a detailed subcellular distribution of fibrils, reinforcing the distinct structural patterns induced by different PFFs.

In conclusion, our findings suggest that αSyn/Tau interactions drive disease-specific structural polymorphs, influencing aggregation kinetics, phosphorylation, and amyloid formation. The ability of hybrid and mixed PFFs to generate distinct inclusion structures underscores their potential role in the molecular heterogeneity of neurodegenerative diseases. These insights advance our understanding of amyloid copathology and may inform therapeutic strategies targeting polymorphic amyloid strains.

## Supplementary Material



## Data Availability

All data are available in the main text or the Supporting Information.

## Notes

Supplementary tables, supplementary figures, and additional experimental details. Data S1–S3: Additional analytical data (PDF)
