# Supplementary material for: Strain-Distinct α‑Synuclein and Tau Cross-Seeding Uncovered by Correlative Approach with Optical Photothermal Infrared Sub-Micron Imaging
Source: J Am Chem Soc. 2025 Jul 29;147(31):27323–40. doi: 10.1021/jacs.5c02811 (PMC12333023; doi:10.1021/jacs.5c02811)
Supplement: Supplementary file 1 [file ja5c02811_si_001.pdf]

Supporting Information for

Strain-distinct α-Synuclein and Tau Cross-seeding Uncovered by Correlative Approach with Optical Photothermal Infrared Sub-Micron Imaging

Xiaoni Zhan[1,2], Wen Li[2,3], Eric Hatterer[4], Jean-Philippe Courade[5], Kristin Piché[6], Oxana Klementieva[7,8]*, Jia-Yi Li[2,3]*

Affiliations

1. Department of Forensic Genetics and Biology, School of Forensic Medicine, China Medical University, Shenyang 110122, China
2. Neural Plasticity and Repair Unit, Department of Experimental Medical Science, Wallenberg Neuroscience Center, Lund University, BMC A10, Lund 22184, Sweden
3. Health Sciences Institute, Key Laboratory of Major Chronic Diseases of Nervous System of Liaoning Province, China Medical University, Shenyang 110122, China
4. Light Chain bioscience Chemin du pré-Fleuri 15, Plan-les-Ouates 1228, Switzerland
5. Discoveric Bio alpha, Bahnhofstrasse 1, Pfäffikon 8808, Switzerland
6. StressMarq Biosciences Inc.,118-1537 Hillside Ave, Victoria BC, V8T 2C1, British Columbia
7. Medical Microspectroscopy Research Group, Department of Experimental Medical Science, Lund University, BMC B10, Lund 22180, Sweden
8. NanoLund, Lund University, Lund 22100, Sweden

* Correspondence:

Jia-Yi Li

Email: lijiayi@cmu.edu.cn; jia-yi.li@med.lu.se

Oxana Klementieva

Email: oxana.klementieva@med.lu.se

Preparation of pre-formed fibrils (PFFs)

The pre-formed fibrils (PFFs) were prepared following a protocol previously published [1]. Pure PFFs were generated from αSyn and Tau monomers. Recombinant human αSyn and human Tau isoforms (0N3R and 2N4R) were purified before use. αSyn PFFs (1 mg/ml) were prepared by constantly agitating αSyn monomers in a thermomixer (1000 rpm at 37°C; Eppendorf) for 5 days of incubation. The hybrid PFFs were produced with equal amounts of αSyn and Tau monomers (1 mg/ml of each) by adding Tau into αSyn solutions before the fibrillization was initiated. Both αSyn and the hybrid PFFs are generated without heparin and are finally prepared in 1X PBS, pH 7.4. Tau PFFs (1 mg/ml) were prepared by mixing equimolar tau with low molecular weight heparin and 2 mM DTT in 100 mM sodium acetate buffer (pH 7.0) and constantly agitated for 5 days (1000 rpm at 37°C). After fibrillization, tau PFFs are transferred into 1× PBS, pH 7.4, to match the buffer conditions of αSyn and hybrid PFFs. The mixed PFFs were freshly prepared by directly mixing αSyn and Tau PFFs before the experiment.

O-PTIR imaging and analysis for PFFs

The sonicated PFFs (1mg/ml, 5µl of each) were airdried on gridded glass coverslips (Ibidi #10816) for further analysis. Brightfield images with a 40× magnification objective of O-PTIR were taken before scanning with 0.78 NA, and an 8 mm working distance Schwarzschild objective. O-PTIR imaging was performed on a mIRage Infrared Microscope (Photothermal Spectroscopy Corp., Santa Barbara, CA, USA). Before measurements, the O-PTIR microscope was purged with N_2 to achieve a 1–2% humidity. The XY position of the IR beam was optimized using recommended points (1730 cm^{-1}, 1410 cm^{-1}, and 1260 cm^{-1}). The Autofocus function was used and built-in Y and Z before starting the background. The spectra were acquired at 2 cm^{-1} points and 100 cm^{-1}/s scanning rate between 800 cm^{-1}–1800 cm^{-1}. The collection parameters were: 1450–1790 cm^{-1} spectral range, in transmission or reflection mode at 2 cm^{-1} spectral resolution. To avoid radiation damage, an APD detector for reflection mode (probe power 0.5% and IR power 58%) and the O-PTIR transmission detector for measurements in transmission mode were used. Spectra were acquired from 5-8 scattered locations of the sonicated pure PFFs resolved in PBS. All of the experiments were reproduced 3 times. The O-PTIR spectra were analyzed following the guidelines provided in the published tutorial [2]. Initially, the spectra were de-glitched using the PTIR Studio software provided by the manufacturer of Mirage™ and then processed using the Preprocess Spectra widget and Peak-fitting function available in Quasar 1.11.1 software [3]. The spectral range was cut to 1600-1700 cm^{-1} to focus on Amide I. Subsequent steps included baseline correction and vector normalization of the spectrum. Analysis was conducted using two different methods: the second derivatives [4] were calculated using a Savitzky-Golay Filter, and peak fitting [5] was based on the peak locations identified in the second derivatives. The peak height intensity and the area under the curve (AUC) were quantitatively measured for each method. The proportions of different amyloid structures in the total composition of peak-fitting were determined and presented in the final results.

References

(1) Pan, L.; Li, C.; Meng, L.; Tian, Y.; He, M.; Yuan, X.; Zhang, G.; Zhang, Z.; Xiong, J.; Chen, G.; et al. Tau accelerates alpha-synuclein aggregation and spreading in Parkinson's disease. *Brain* **2022**, *145* (10), 3454-3471. DOI: 10.1093/brain/awac171.

(2) Prater, C. B.; Kansiz, M.; Cheng, J. X. A tutorial on optical photothermal infrared (O-PTIR) microscopy. *APL Photonics* **2024**, *9* (9), 091101. DOI: 10.1063/5.0219983.

(3) Toplak, M.; Read, S. T.; Sandt, C.; Borondics, F. Quasar: Easy Machine Learning for Biospectroscopy. *Cells* **2021**, *10* (9). DOI: 10.3390/cells10092300.
(4) Calero, M.; Gasset, M. Fourier transform infrared and circular dichroism spectroscopies for amyloid studies. *Methods Mol Biol* **2005**, *299*, 129-151. DOI: 10.1385/1-59259-874-9:129. Usoltsev, D.; Sitnikova, V.; Kajava, A.; Uspenskaya, M. Systematic FTIR Spectroscopy Study of the Secondary Structure Changes in Human Serum Albumin under Various Denaturation Conditions. *Biomolecules* **2019**, *9* (8). DOI: 10.3390/biom9080359.
(5) Zucchiatti, P.; Mitri, E.; Kenig, S.; Bille, F.; Kourousias, G.; Bedolla, D. E.; Vaccari, L. Contribution of Ribonucleic Acid (RNA) to the Fourier Transform Infrared (FTIR) Spectrum of Eukaryotic Cells. *Anal Chem* **2016**, *88* (24), 12090-12098. DOI: 10.1021/acs.analchem.6b02744 From NLM Medline. Wei, W.; Hu, W.; Zhang, X. Y.; Zhang, F. P.; Sun, S. Q.; Liu, Y.; Xu, C. H. Analysis of protein structure changes and quality regulation of surimi during gelation based on infrared spectroscopy and microscopic imaging. *Sci Rep* **2018**, *8* (1), 5566. DOI: 10.1038/s41598-018-23645-3.

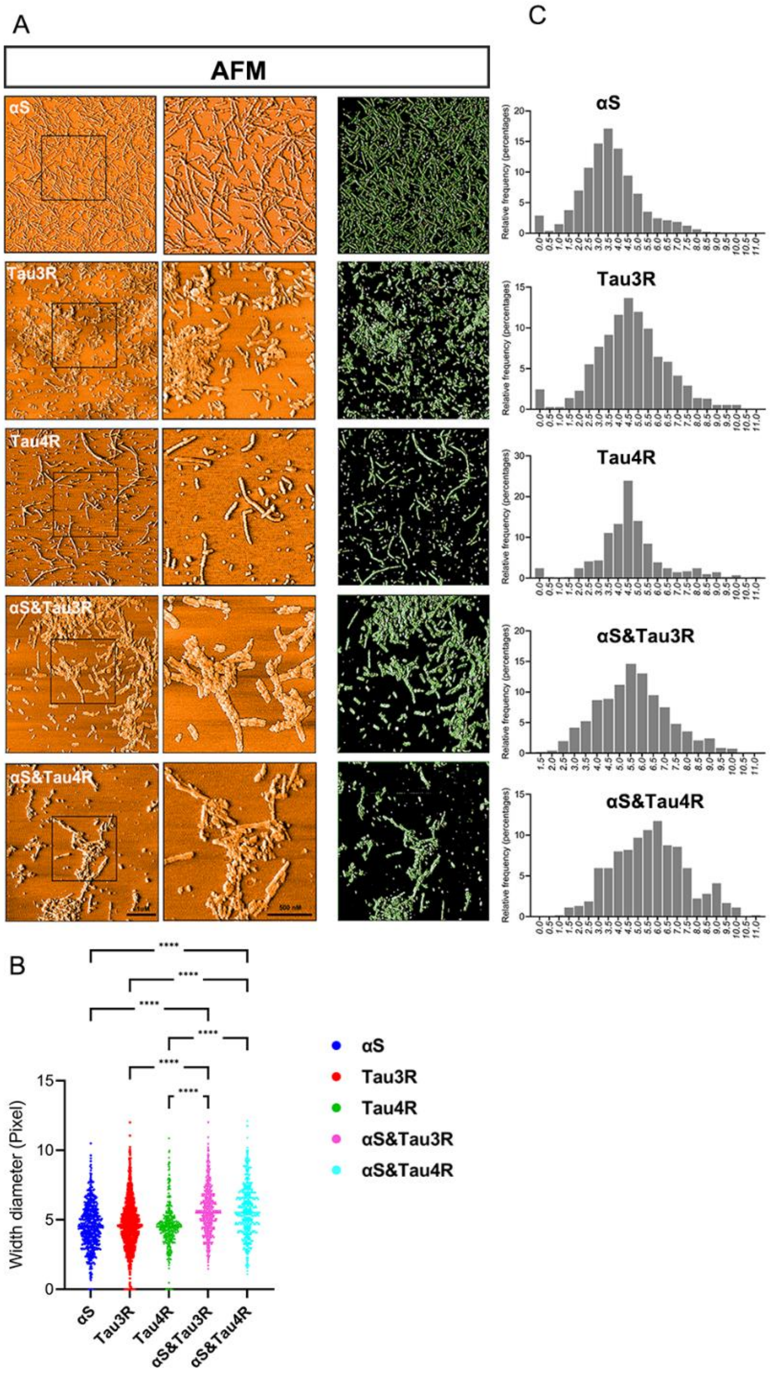

Fig. S1. Morphological characterization of αS/Tau PFFs using AFM.

(A) AFM images display the morphologies of pure αS, Tau PFFs and their co-polymers (hybrid PFFs), Scale bar= 1 um (first column), and 500 nm for the zoomed images (second column). Masks highlighting the fibrils analyzed are shown adjacent to each image to delineate the regions of interest for detailed assessment (third column). **(B)** The average width diameters of different fibrils are presented in a dot plot. Statistical significance was assessed using one-way ANOVA, with a significance level set at $P < 0.05$. **(C)** The size distribution of fibril diameters across different fibrils are displayed in a histogram.

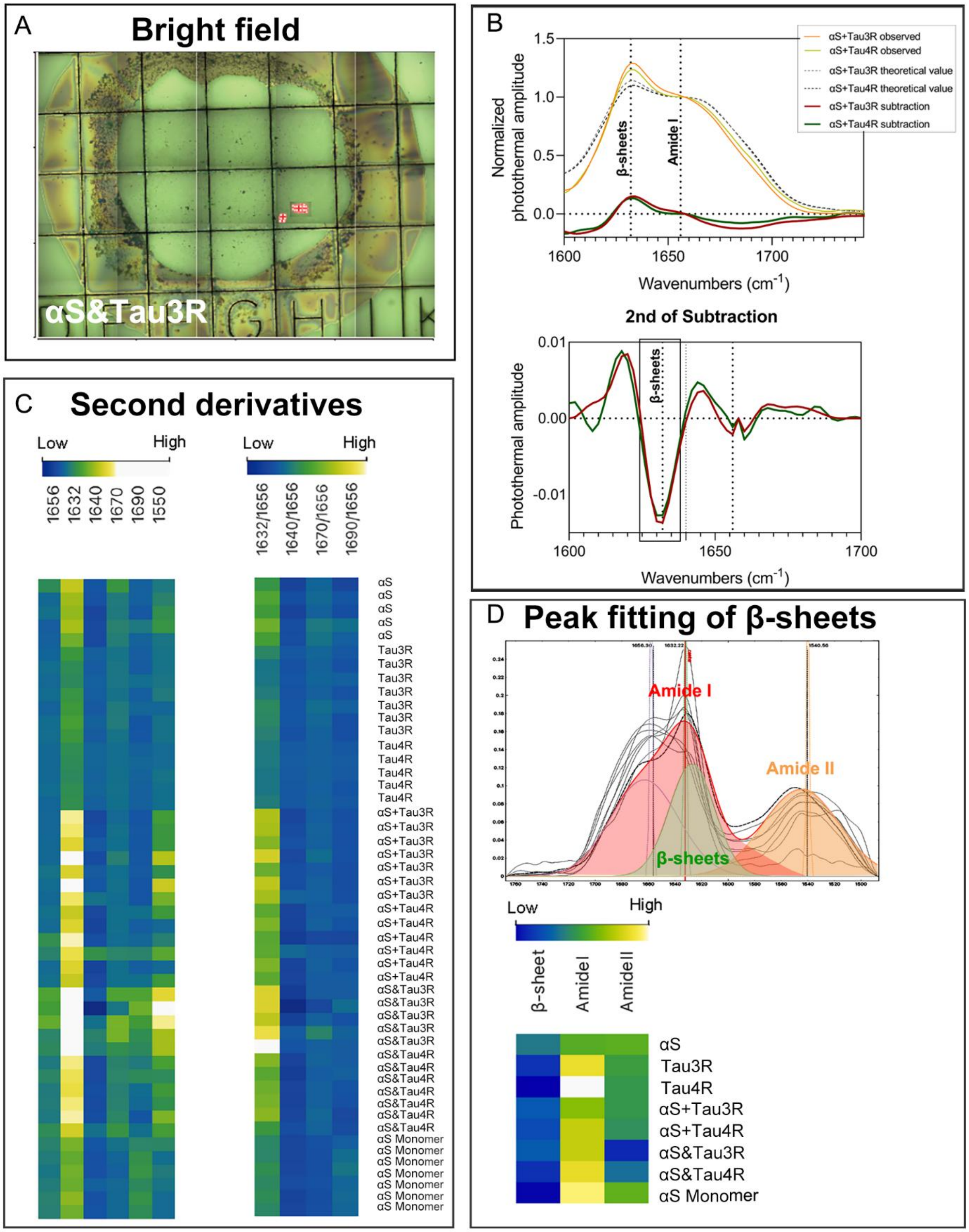

Fig. S2. Heatmaps of amyloid structure intensity of different PFFs.

(A) Brightfield images of air-dried PFFs on the coverslips. **(B)** The observed and theoretical O-PTIR spectrum of αS+Tau3R and αS+Tau4R are plotted in the same axis after normalizing to

the total protein (1658 cm^{-1}). **αS+Tau3R theoretical**=1/2 αS+1/2 Tau3R (grey dotted line); **αS+Tau4R theoretical** =1/2 αS+1/2 Tau4R (black dotted line); **αS+Tau3R subtraction** (dark red) = αS+Tau3R observed–αS+Tau3R theoretical; **αS+Tau4R subtraction** (dark green) **=** αS+Tau4R observed–αS+Tau4R theoretical. The second derivatives of **subtracted spectrum** demonstrate an increase in β-sheets component (around 1630 cm^{-1}). **(C)** Heatmaps of the peak intensity from original O-PTIR spectra alongside normalized ratios, deciphered using the second derivatives method, corresponding to the amyloid band structures of each PFF sample. **(D)** Schematic for peak fitting approach to resolve different components (Amide I and II, and β-sheets) in the PFFs. The area under the curve (AUC) of the average spectrum from different PFFs are quantified and displayed in the heatmap.

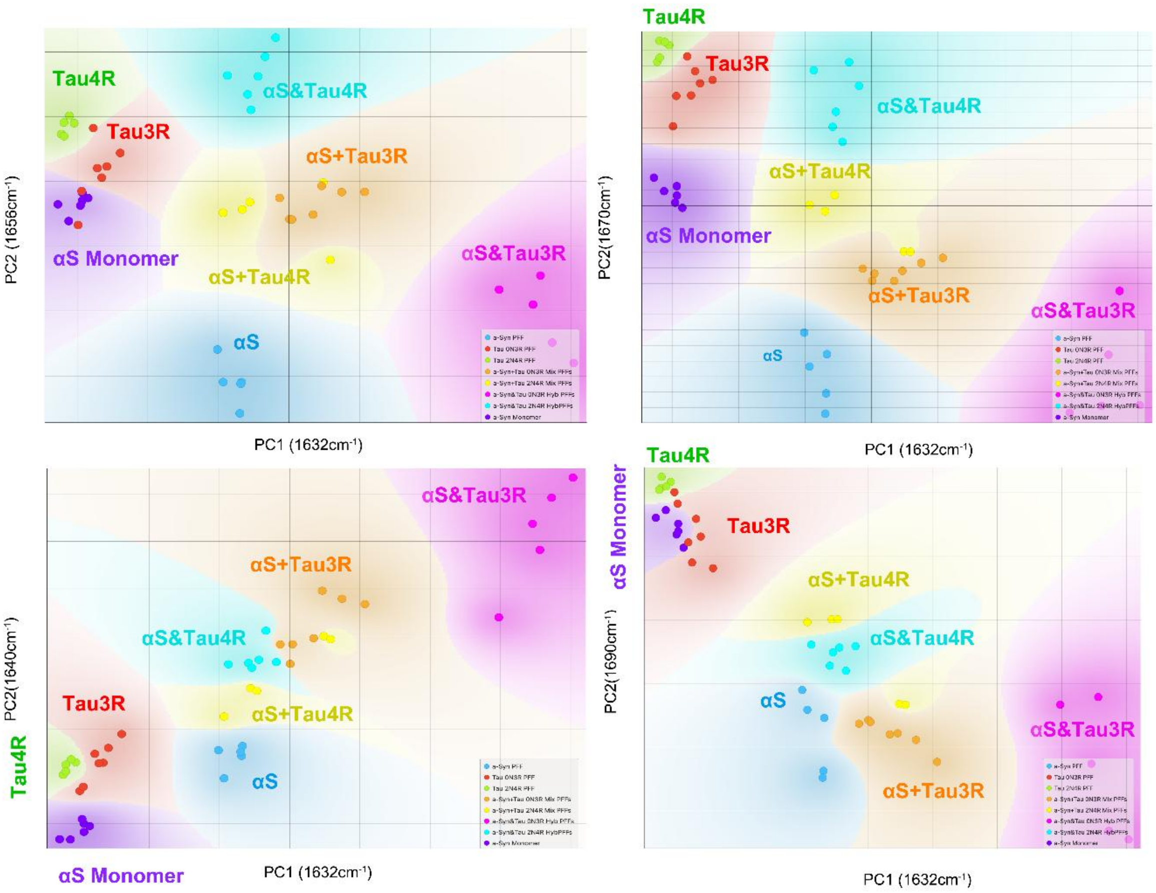

Fig. S3. Principal component analysis of different PFFs.
Principal Component Analysis of eight PFFs is plotted with the wave numbers of the bands corresponding to the amyloid structures as the axis. 1632 cm^{-1}: (major β-sheet parallel); 1640 cm^{-1} (random coils); 1670 cm^{-1} (β-turns); 1690 cm^{-1} (minor β-sheet anti-parallel).

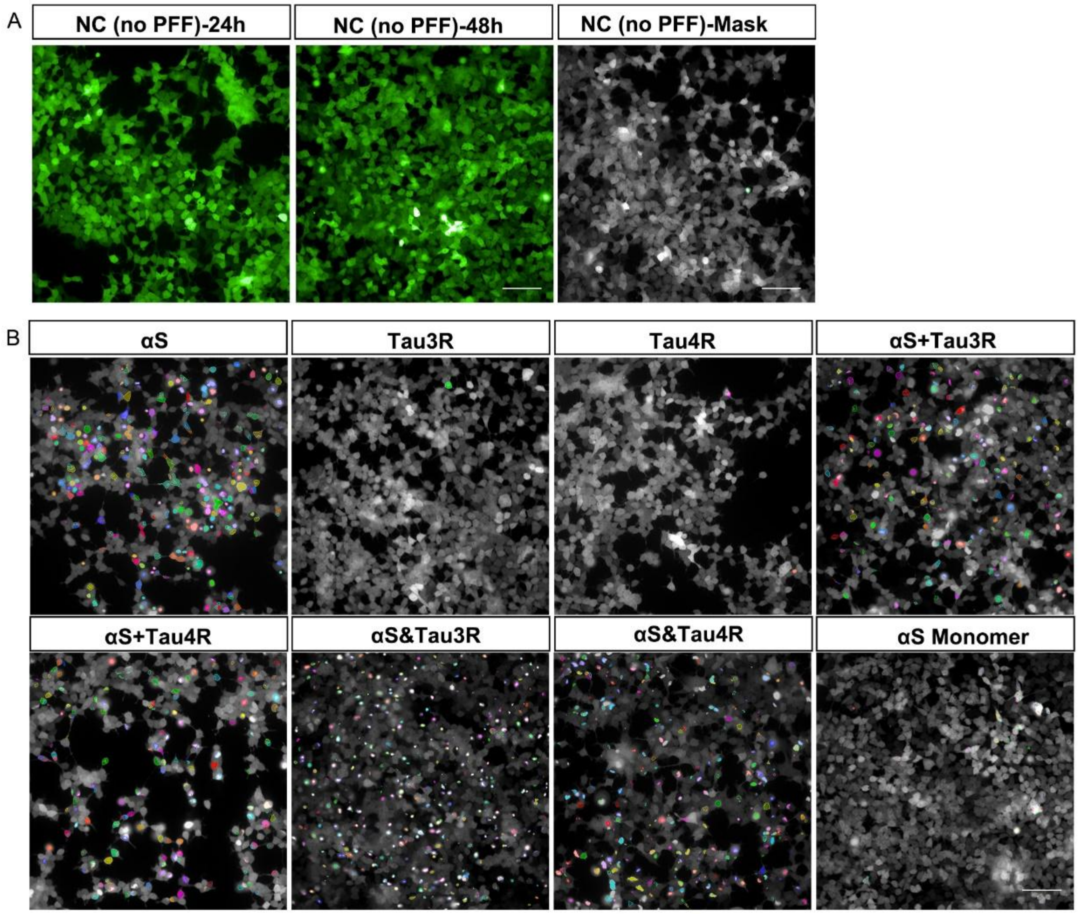

Fig. S4. Mask mode for aggregate analyses with the Operetta CLS system.
(A) No visible aggregates in α-Syn-A53T-GFP HEK cells are observed in the NC group at 24 h and 48 h, **(B)** The intracellular aggregates above the thresholds are automatically selected and marked with rainbow outlines (colors have no relevance to intensity) in the images taken 48 h after seeding with different PFFs. No visible inclusions were found in cells treated with Tau or αS Mono. Scale bars= 10 µm.

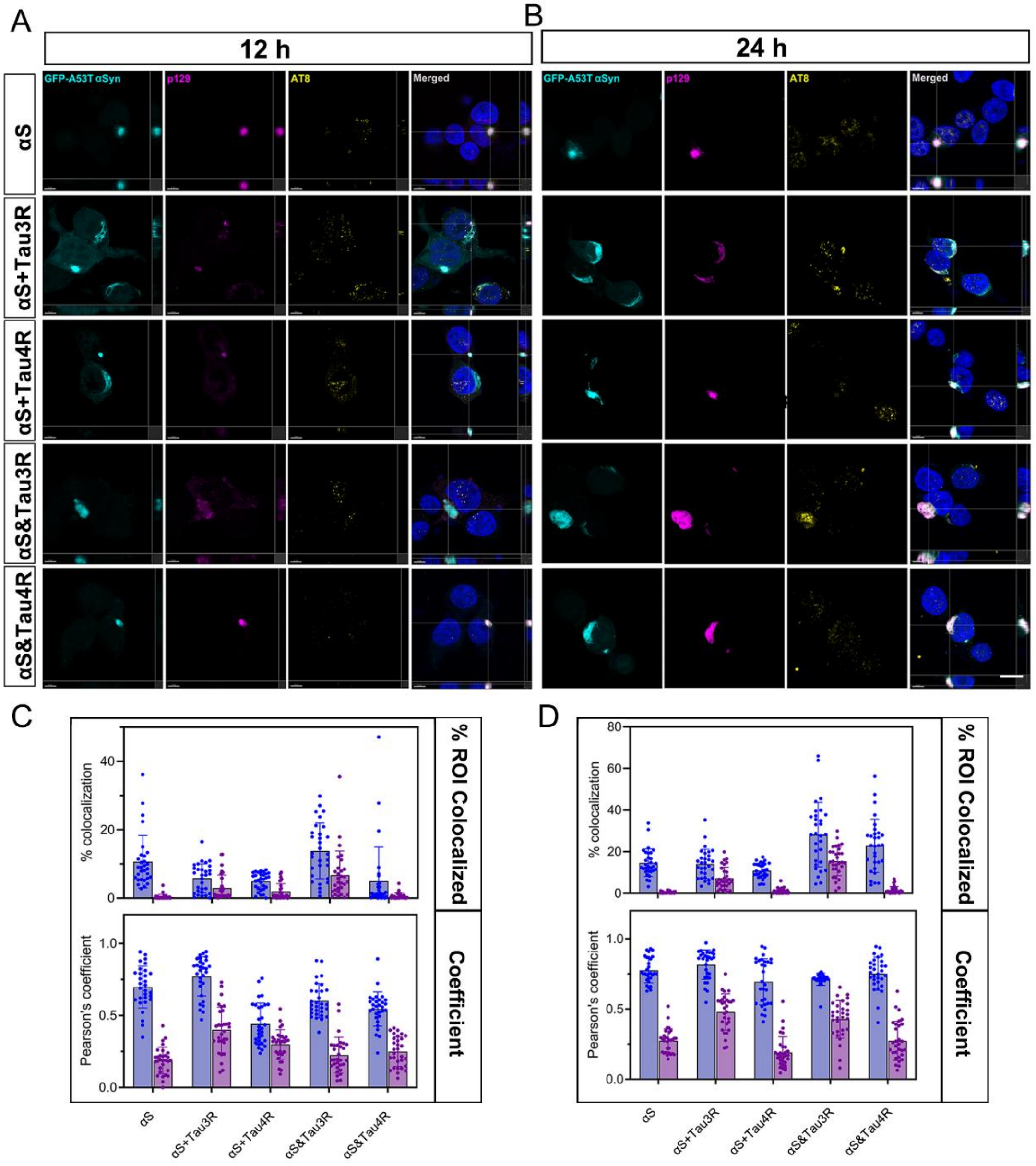

Fig. S5. Increased α-Syn and Tau levels of phosphorylation in αSyn-GFP inclusions as time extends.

(A-B) Increased phosphorylated α-Syn and Tau levels in the inclusions are observed during the first 24 h in the confocal images of p-129 and AT8 co-labeling. Significant Tau phosphorylation is indicated at 24 h. Scale bar= 10 µm, **(C-D)** Colocalization of p-129 and AT8 seeded at 12 and 24 h are evaluated with % colocalization and Pearson's coefficient. Bar plots are shown as mean ± s.d.

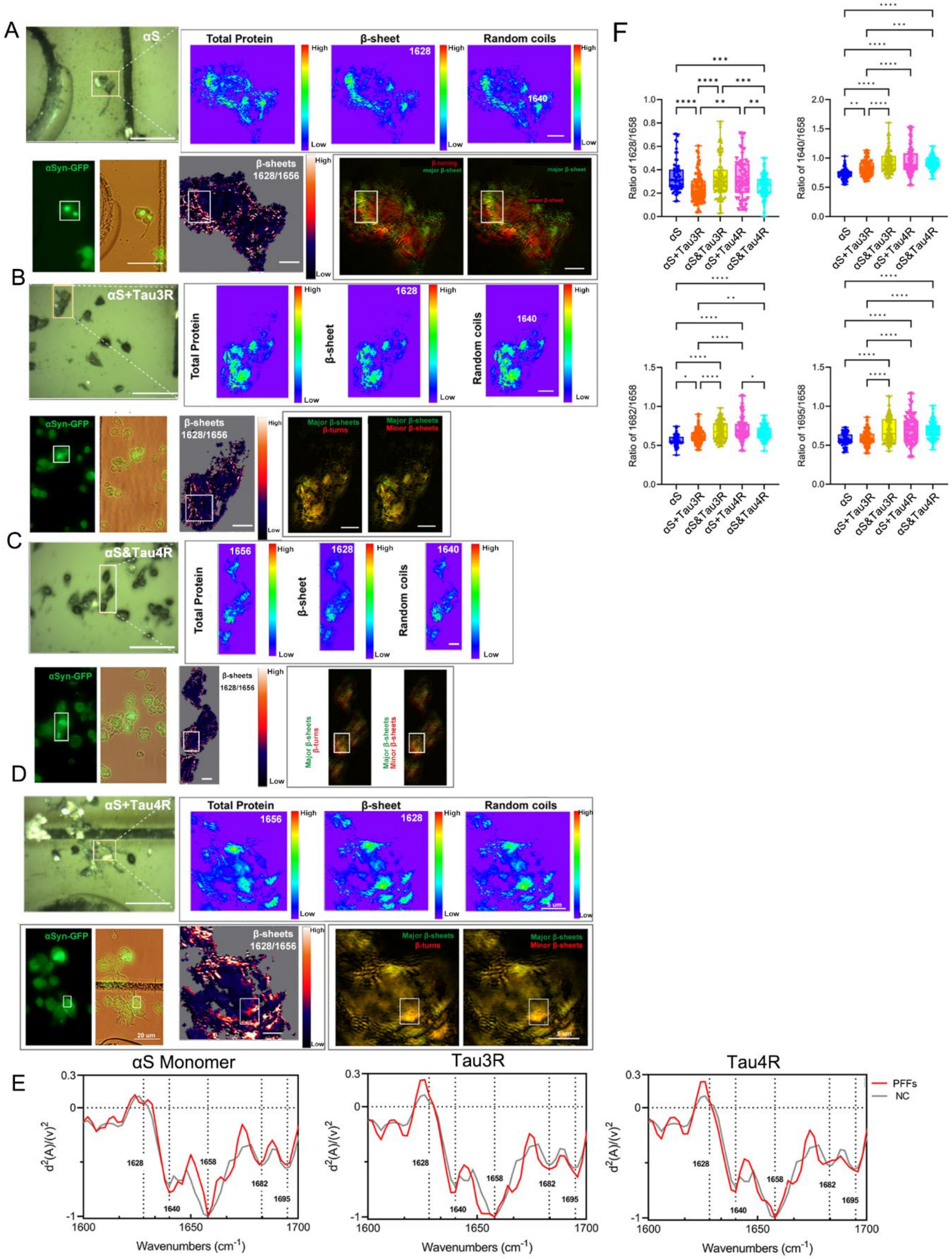
A
αS
Total Protein
β-sheet
Random coils
1628
1640
High
Low
αSyn-GFP
β-sheets
1628/1656
β-turning
major β-sheet
minor β-sheet
B
αS+Tau3R
Major β-sheets
β-turns
Minor β-sheets
C
αS&Tau4R
1656
D
αS+Tau4R
20 um
5 um
E
αS Monomer
Tau3R
Tau4R
PFFs
NC
d2(A)/(v)2
Wavenumbers (cm-1)
1600
1650
1700
1658
1682
1695
F
Ratio of 1628/1658
Ratio of 1640/1658
Ratio of 1682/1658
Ratio of 1695/1658
αS
αS+Tau3R
αS&Tau3R
αS+Tau4R
αS&Tau4R

Fig. S6. Amyloid structures of αSyn aggregates are induced by different PFFs.

(A-D) Correlative analysis of fluorescence and O-PTIR microspectroscopy to identify the same aggregates in cells seeded with αS **(A)**; αS+Tau3R **(B)**; αS&Tau4R **(C)**; αS+Tau4R **(D)**. Single energy O-PTIR images and overlays displays distributions of the subcellular distribution of secondary structures: 1658 cm^{-1} (α-helix/ total proteins); 1628 cm^{-1} (β-sheet major parallel); 1640 cm^{-1} (random coils). The same layout of the correlative analysis of fluorescence and O-PTIR as Fig 4. Scale bars are 20 μm in the brightfield and fluorescent images, and 5 μm in the O-PTIR images, **(E)** Compared to NC groups average and normalized second derivatives of O-PTIR spectra from cells without visible inclusions. No evident band for 1628 cm^{-1} was found for the cells seeded with two Tau or αS Monomer, **(F)** Statistical analysis of protein aggregation measured as the average of the protein aggregation ratios of β-sheet structures (1628 cm^{-1}), random coils (1640 cm^{-1}), β-turns (1682 cm^{-1}), minor β-sheet (1695 cm^{-1}) to α-helix structures (1658 cm^{-1}) in cells with aggregates.

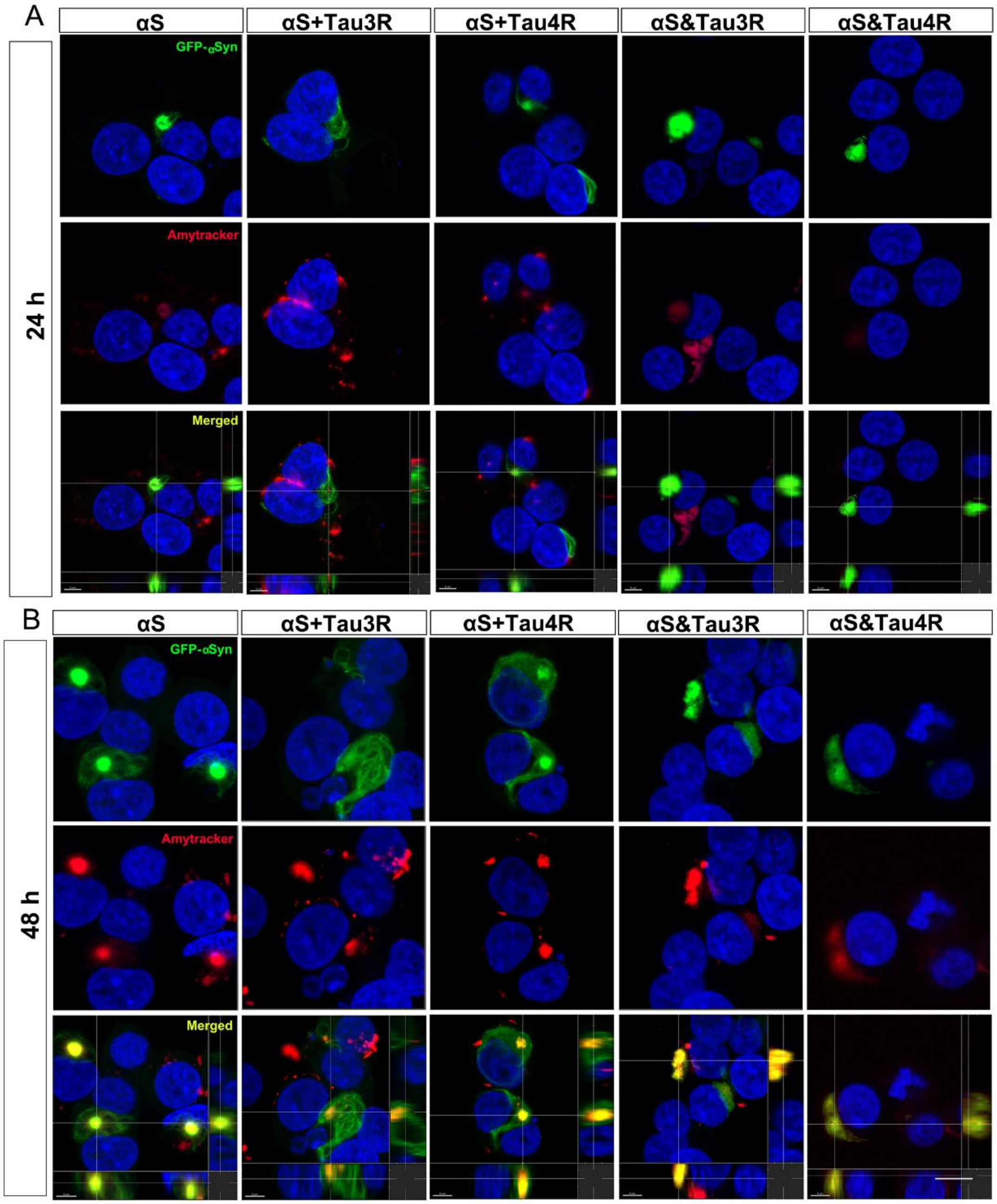

Fig. S7. Confocal images of Amytracker labeled αSyn aggregates in cells seeded with various PFFs at 24 h and 48 h.

Amyloids were labeled with the fibril-specific dye Amytracker (red), the green color corresponds to α-Syn-GFP aggregates in cells. Confocal images of cells at 24 h **(A)**, 48 h **(B)** post PFFs seeding indicated the morphology and subcellular location of the amyloid structures. Scale bar=10 μm.

Table S1.

Nomenclature of different PFFs in the study

Abbreviation	Content of PFFs
αS	αSyn PFF
Tau3R	Tau 0N3R
Tau4R	Tau 2N4R
αS+Tau3R	αSyn+ Tau 0N3R Mixed PFFs (1:1 mixed)
αS+Tau4R	αSyn+ Tau 2N4R Mixed PFFs (1:1 mixed)
αS&Tau3R	αSyn& Tau 0N3R Hybrid PFFs (1:1 hybrid)
αS&Tau4R	αSyn& Tau 2N4R Hybrid PFFs (1:1 hybrid)

Table S2.

Summary of infrared spectra peak positions for second derivatives analysis of the PFFs and cells

Wavenumbers and Ratios (cm^{-1})	Amyloid Structure
1656	C=O stretching, Amide I/α-helix
1550	C-N stretching; N-H bending, Amide II
1632	Major β-sheet (Parallel)
1642	Random coils (unordered)
1670	β-turns
1690	Minor β-sheet (Anti-parallel)

Table S3.

Secondary structures intensity resolved from the average spectrum of different PFFs

PFF Samples	Secondary Structures*			
	Major β-sheets	Random coils	β-turns	Minor β-sheets
αS	13.7	3.7	7.7	6.0
Tau3R	9.6	5.5	6.9	5.7
Tau4R	8.6	6.1	6.8	6.2
αS+Tau3R	18.8	5.2	6.7	5.1
αS+Tau4R	17.6	5.2	7.1	6.7
αS&Tau3R	31.4	3.3	8.3	7.3
αS&Tau4R	18.1	4.2	8.0	6.5
αS Monomer	12.6	5.0	6.3	6.5

*Peak intensity×10^{-3} (second derivatives)

Data in table is corresponding to the heatmap in Fig. 2

Table S4.

Summary of peak ratios used for second derivatives analysis of the cell

Ratios	Amyloid Structures
1628 cm^{-1}/1658 cm^{-1}	Major β-sheet (Parallel)
1640 cm^{-1}/1658 cm^{-1}	Random coils (unordered)
1682 cm^{-1}/1658 cm^{-1}	β-turns
1695 cm^{-1}/1658 cm^{-1}	Minor β-sheet features (Anti-parallel)

Table S5.

Peak intensity ratios of secondary structures from the average spectrum in cells with different aggregates

Cell Samples	Secondary Structures*			
	Major β-sheets	Random coils	β-turns	Minor β-sheets
αS	0.342	0.731	0.564	0.576
αS+Tau3R	0.242	0.826	0.616	0.587
αS+Tau4R	0.335	0.940	0.687	0.696
αS&Tau3R	0.352	0.945	0.712	0.709
αS&Tau4R	0.260	0.913	0.668	0.693

*Intensity ratio is rounded to three decimal places

Data in table is corresponding to the heatmap in Fig. 5

Table S6.

Average peak intensity ratios of secondary structures in α-Syn aggregates induced by different PFFs

Cell Samples	Segment	Secondary Structures*			
		Major β-sheets	Random coils	β-turns	Minor β-sheets
αS	Core	0.614	0.793	0.606	0.681
	High	0.413	0.803	0.596	0.662
	Low	0.317	0.792	0.567	0.647
αS+Tau3R	Core	0.352	0.799	0.540	0.591
	High	0.402	0.72	0.507	0.583
	Low	0.274	0.719	0.555	0.591
αS+Tau4R	Core	0.423	0.823	0.581	0.644
	High	0.419	0.797	0.566	0.610
	Low	0.351	0.825	0.573	0.630
αS&Tau3R	Core	0.486	0.712	0.561	0.629
	High	0.435	0.634	0.497	0.548
	Low	0.185	0.717	0.529	0.598
αS&Tau4R	Core	0.411	0.774	0.532	0.600
	Low	0.326	0.745	0.515	0.572

*Intensity ratio is rounded to three decimal places

Data in table is corresponding to the heatmap in Fig. 6

Data S1.

Multiple comparisons of seeding effects as a function of time.

Quantifications of % Cell with aggregates, aggregates area per well and GFP-regional intensity. Significance is decided by adjusted $P < 0.05$.

Data S1. xlsx

Data S2.

Multiple comparisons of αSyn and Tau phosphorylation.

Quantifications of αSyn (p-129) and Tau (AT8) intensity and their overlaps in αSyn inclusions in each group, including %colocalization and Pearson's coefficient at 12, 24, 48 h and solubility of TX-100, Sarkosy and PK resistance at 48 h. Significance is decided by adjusted $P < 0.05$.

Data S2. xlsx

Data S3.

Multiple comparisons of secondary structure intensity of various PFFs.

Data S3. xlsx
